# Adaptive oligodendrogenesis regulates blood-hypothalamus barrier permeability, hypothalamic glucose sensing and systemic glucose homeostasis in male mice

**DOI:** 10.1016/j.molmet.2026.102428

**Published:** 2026-08-06

**Authors:** Sophie Buller, Emily O. Staricoff, Christine Riches, Anthony Tsang, Giada Giavara, Masa Josipovic, Kentaro Ikemura, Gabriel Opoku, Ikumi Sato, Satoshi Hirohata, Saskia Stenzel, Stuart G. Nayar, Marta Ramos Vega, Jacob Hecksher-Sørensen, Sebastian Timmler, Georgina K.C. Dowsett, Brian Y.H. Lam, Giles S.H. Yeo, Kimberly M. Alonge, Huiliang Li, William D. Richardson, Mark L. Evans, Clemence Blouet

**Affiliations:** 1 Institute of Metabolic Science Metabolic Research Laboratories, University of Cambridge, Cambridge, CB2 0QQ, UK; 2 MRC Metabolic Diseases Unit, Institute of Metabolic Science Metabolic Research Laboratories, University of Cambridge, Cambridge, CB2 0QQ, UK; 3 Department of Medical Technology, Graduate School of Health Sciences, Okayama University, Okayama, 700-8558, Japan; 4 Wolfson Institute for Biomedical Research, University College London, London, WC1E 6BT, UK; 5 GUBRA, Hørsholm, Denmark; 6 Cambridge Stem Cell Institute, University of Cambridge, CB2 0AW, UK; 7 Diabetes Institute, University of Washington, Seattle, WA, 98109, USA

**Keywords:** Oligodendrogenesis, Median eminence, Blood-hypothalamus barrier, ADAMTS4, Glucose sensing, Hypoglycaemia

## Abstract

**Objective:**

Brain glucose sensing is critical for survival during hypoglycaemia, yet how glucose-sensing neurons access circulating glucose concentrations to maintain glucose homeostasis remains poorly understood. Here we tested the hypothesis that adult oligodendrogenesis in the median eminence (ME) is responsive to changes in blood glucose levels and contributes to hypothalamic glucose sensing through regulation of the blood-hypothalamus barrier.

**Methods:**

We used glycemic challenges and hypoinsulinaemic clamp studies to identify the effect of systemic changes in glycaemia on hypothalamic oligodendrocyte lineage cells. We used conditional knockout mouse models to dissect the respective contributions of adult oligodendrogenesis and new myelin formation to glucose homeostasis in adult male mice. Analyses combined immunofluorescence, serial electron microscopy, whole-brain tissue clearing, and transcriptomic analyses.

**Results:**

We found that adult oligodendrogenesis in the median eminence (ME) is modulated by changes in circulating glucose levels and rapidly upregulated by hypoglycaemia. Genetic blockade of new oligodendrocyte production in adult male mice impairs the regulation of glucose homeostasis, the integrity of the ME blood-hypothalamus barrier, and hypothalamic glucose sensing. Unexpectedly, functional integrity of adult-formed myelin is not required for the maintenance of glucose homeostasis. Instead, we show that blockade of adult oligodendrogenesis disrupts hypothalamic expression of A disintegrin and metallopeptidase with thrombospondin motifs 4 (ADAMTS4), a metallopeptidase whose brain expression is restricted to the oligodendrocyte lineage and whose ME expression requires ongoing adult oligodendrogenesis. We show that ADAMTS4 regulates hypothalamic perineuronal net deposition, vascular permeability and glucose sensing. Finally, we show that ME ADAMTS4 expression is regulated by changes in peripheral glycaemia and is dysregulated in diabetes, providing a mechanism by which ME oligodendrocytes contribute to the regulation of glucose homeostasis.

## Highlights

• Hypoglycaemia rapidly increases oligodendrogenesis in the median eminence.
• Adult oligodendrogenesis is required for the maintenance of glucose homeostasis.
• New compact myelin is dispensable for glucose homeostasis regulation.
• Adult oligodendrogenesis regulates blood-hypothalamus barrier permeability.

## 1 Introduction

The brain plays a crucial role in the response to hypoglycaemia by coordinating behavioural and neuroendocrine counterregulatory responses necessary for a rapid return to euglycaemia [1]. This essential survival response is mediated by neurocircuitries distributed between the hindbrain and mediobasal hypothalamus, enriched in hypoglycaemia-activated neurons [[2], [3], [4]]. The efficiency of brain glucose sensing to restore systemic glucose homeostasis relies on the ability of glucose-sensing circuits to rapidly detect and respond to changes in circulating glucose concentrations with great accuracy and sensitivity. Although the blood–brain barrier shields most of the brain from fluctuations in circulating glucose, glucose-sensing neurons are concentrated near circumventricular organs. In these regions, a modified blood–brain barrier creates a distinct microenvironment in which extracellular glucose levels closely reflect systemic glucose availability [5].

The median eminence (ME) is a circumventricular organ located at the base of the hypothalamus. Fenestrated capillaries within the ME disrupt the local blood–brain barrier. Instead, a diffusion barrier between the ME and the adjacent parenchyma of the arcuate nucleus of the hypothalamus (ARH) regulates local access of blood-borne signals [5]. The ME diffusion barrier exhibits a high degree of plasticity under physiological conditions. For example, it is rapidly remodelled in response to energy or glucose deficit [6], altering diffusion properties of the blood-hypothalamus barrier, thereby modulating the access of circulating signals to the adjacent hypothalamic parenchyma [5]. However, the cellular and molecular mechanisms underlying this plasticity remain incompletely understood.

Oligodendrocyte progenitor cells (OPCs) persist throughout adult life and form a distributed, self-renewing population of glial progenitors that continue to generate new oligodendrocytes in the mature brain [[7], [8], [9], [10], [11]]. Adult oligodendrogenesis is recruited by experience, learning and motor training, and the resulting new myelin is required for long-term memory and for the refinement of circuit timing [[12], [13], [14], [15], [16]]. OPC differentiation also occurs outside classical white-matter tracts, including in the hypothalamus, where ME OPCs proliferate and differentiate at unusually high rates in the healthy adult brain [[17], [18], [19], [20], [21]]. Recent work links ME OPC differentiation to systemic energy availability [20], but the specific nutritional cues that regulate it remain unknown.

Here we tested the hypothesis that ME oligodendrogenesis is responsive to changes in blood glucose levels and participates in the structural changes occurring within the ME to facilitate hypothalamic glucose sensing.

## 2 Methods

### 2.1 Animals

All animal experiments were performed on male adults and in accordance with the UK Home Office regulations under the Animals (Scientific Procedures) Act (1986) and with the approval of the University of Cambridge Animal Welfare and Ethics Review Board. Animals were group-housed in a specific pathogen free facility and maintained on a standard 12-hour light/dark cycle (lights on 7:00–19:00) at 22 °C with ad libitum access to water and standard laboratory chow (SAFE R105, SAFE Complete Care Competence, Rosenberg, Germany) unless otherwise stated. All experiments were performed on male mice starting from postnatal day 60 (P60). C57BL/6J mice were obtained from Charles Rivers Laboratories (Saffron Walden, UK). Conditional deletion of *Myrf* or *Mbp* from adult OPCs used the tamoxifen-inducible *Pdgfra*-CreERT2 driver line. Floxed *Myrf* (*Myrf*^*fl/fl*^) [22] and floxed *Mbp* (*Mbp*^*fl/fl*^; S.G.N., H.L. and W.D.R. unpublished) mice were provided by Professor William Richardson at University College London and crossed to *Pdgfra*-CreERT2;*Rosa26R*-eYFP mice to generate *Pdgfra*-CreERT2;*Rosa26R*-eYFP;*Myrf*^*fl/fl*^ and *Pdgfra*-CreERT2;*Rosa26R*-eYFP;*Mbp*^*fl/fl*^ animals, hereafter referred to as Myrf^fl/fl^ and Mbp ^l/fl^, respectively. Cre-negative littermates (*Myrf*^+/+^ and *Mbp*^+/+^), generated from the same *Pdgfra*-CreERT2; fl/+ × fl/+ crosses and therefore sharing the same mixed genetic background, served as controls throughout. This *Mbp*^*fl/fl*^ line is structurally and phenotypically similar to that described by Meschkat et al. [23] but was conceived and made independently. Tissues from *Adamts4* knockout (*Adamts4*^−/−^) mice were obtained from Professor Satoshi Hirohata at Okayama University.

### 2.2 Tamoxifen administration and preparation

Tamoxifen (Sigma) was prepared in corn oil (Sigma) by sonication at 37 °C at 30 mg/ml prior to administration by oral gavage at 300 mg/kg of body weight on 4 consecutive days.

### 2.3 Glucose challenges in C57BL/6J mice

C57BL/6J mice were randomly allocated to groups and received four intraperitoneal (i.p.) injections of BrdU (Sigma; 50 mg/kg - prepared in sterile saline at 5 mg/ml) within a 24-hour period starting at ZT5 and ending at ZT4 on the following day. Mice were fasted for 4 h prior to the fourth injection, when mice received BrdU alone (vehicle) or in combination with glucose (2 g/kg), insulin (0.75 U/kg) or 2DG (250 mg/kg). One hour later a blood glucose reading was obtained from the tail vein at ZT5 immediately prior to sacrifice using an AlphaTrak 2 handheld glucometer (Precision Xtra; MediSense).

### 2.4 Streptozotocin-induced diabetes in diet-induced-obese mice

C57BL/6J mice were fed a high fat diet with 60% kilocalories obtained from fat (60% HFD; Research Diets D1492i) from 8 weeks of age for 8 weeks. After 8 weeks, animals were randomly allocated to groups and a baseline blood glucose reading was obtained from the tail vein using an AlphaTrak 2 handheld glucometer prior to ip administration of either vehicle (saline; 10 ml/kg) or streptozotocin (STZ, Sigma; 100 mg/kg). Metabolic phenotyping tests were performed 4 weeks after vehicle or STZ administration.

### 2.5 Hyperinsulinaemic clamp paradigm

In brief, C57BL/6J mice underwent surgery under isoflurane anaesthesia using a low-flow anaesthesia delivery system (SomnoSuite® - Kent Scientific Corporation) to implant vascular catheters into the left carotid artery and right jugular vein. Vascular lines were exteriorised behind the nape of the neck using a vascular access button (Instech Laboratories) allowing post-operative group-housing.

On study days, animals were fasted from ZT1 and vascular lines flushed and connected via a tether and low-torque dual channel swivel to allow handling-free intravenous infusion and arterial blood sampling. After acclimatization, starting around midday (ZT5), a non-primed continuous intravenous infusion (10 mU/kg/min) of rapid-acting insulin (NovoRapid, Novo Nordisk diluted in Bovine Serum Albumin, Sigma–Aldrich) was delivered together with a simultaneous infusion of dextrose (20% (w/v) or 50% glucose (w/v)- Fresenius Kabi and Hameln pharma respectively). Arterial samples to measure whole blood glucose concentrations were taken every 5–15 min and analysed using a handheld blood glucose meter (Accu-Chek® Aviva, Roche) to minimize blood volume sampling. Additional samples were taken periodically for comparison with plasma glucose concentrations determined biochemically using an Analox Analyser (GM9 Glucose Analyser, Analox Instruments). Dextrose infusion rates were adjusted to achieve target ranges of ≤3.9, 6–8 and ≥15.0 mmol/L for hypoglycaemia, euglycaemia and hyperglycaemia respectively. Clamps were 75 min in length, to allow roughly 30 min to reach the target glycaemic level, and then 45 min in a steady state. If necessary, clamps were extended until blood glucose had been stable within the target range for at least 40 min. Plasma samples for insulin analysis were collected at baseline and at the end of the clamp. Upon completion of the clamp, mice immediately underwent perfusion fixation.

### 2.6 Metabolic tolerance tests

Mice were moved to a procedure room at ZT1, singly housed and fasted in clean cages with home cage enrichment. A baseline blood glucose reading was taken from the tail vein using a handheld glucometer at ZT5. Mice were administered an oral gavage of glucose (2 g/kg; Sigma) for an OGTT, an ip injection of glucose (1 g/kg; Sigma) for an ipGTT or an ip injection of insulin (0.75 U/kg; Actrapid, Novo Nordisk) for an ITT at ZT6 and blood glucose measured 10, 20, 30, 60, 90 and 120 min later. For OGTTs, additional blood samples were obtained at baseline and 20 min after glucose administration for measurement of plasma insulin. At the end of the test, mice were returned to home cages and refed.

### 2.7 Dehydration paradigm

Water was removed from the animals’ home-cage immediately prior to the dark onset (ZT12). Plasma samples were obtained at baseline and after 12 h water deprivation (ZT0) for measurement of plasma osmolarity using an Osmomat 3000 (Gonotec). Following plasma sampling, water was returned to the home cage.

### 2.8 2DG studies

Glucoprivic feeding and hyperglycaemia were assessed via crossover studies. For all experiments, mice were moved to a procedure room, singly housed, and fasted at ZT1 prior to i.p. injection of either saline (Vetivex) or 2DG (250 mg/kg; Sigma) at 10 ml/kg of body weight at ZT5. For food intake studies, animals were provided with standard chow diet 30 min after saline or 2DG administration and food intake measured at 30, 60, 90, 120 and 180 min as loss of food weight in grams. For blood glucose measurements, blood glucose readings were obtained from the tail vein using a handheld glucometer 0, 15, 30, 60, 90 and 120 min after saline or 2DG injection. For measurement of counter-regulatory hormones, plasma samples were collected 30 min after saline or 2DG administration. For assessment of 2DG-induced cFos expression, mice were fasted from ZT1, administered saline or 2DG at ZT5 as described above and underwent perfusion-fixation 90 min later.

### 2.9 Plasma analyses

Whole blood samples were obtained from the tail vein into heparinised capillary tubes and kept on ice until centrifugation with a Haematospin 1300 (Hawksley) to obtain plasma. Glucagon was measured using an enzyme-linked immunosorbent assay (Mercodia AB; product code 10-1271-01). Insulin was measured using the Mouse/Rat Insulin Kit on the MesoScale Discovery immunoassay platform (MesoScale Delivery; product code K152BZC-3). ACTH was measured by sandwich immunoassay using the MilliPlex MAP Mouse Pituitary Magnetic Bead Panel Kit (Merck Millipore; product code MPTMAG-49K). Corticosterone was measured using the Corticosterone EIA Kit (ImmunoDiagnosticSystems; product code AC14F1).

### 2.10 ADAMTS4 injection paradigm

Surgical procedures were carried out in single-housed C57BL/6J mice under isoflurane anaesthesia that received Metacam prior to surgery. Steel guide cannula (Plastics One) were stereotactically implanted 1 mm above the ME-ARH region (A/P: 1.3 mm, D/V: 5.0 mm, lateral: +0.2 mm relative to Bregma) and secured using Loctite glue and dental cement (Fujicem). All mice were acclimatised to handling and the brain injection procedure during the one-week post-operative period. Following recovery from surgery, injections were performed in a crossover manner so that all mice received aCSF on one week and one week later received ADAMTS4. Where injections were repeated, mice were given at least a 4-week wash out period between ADAMTS4 and aCSF injections. On the day of injection, mice were moved to a procedure room at ZT1 and fasted in clean cages with home cage enrichment. Bilateral injections of aCSF (Tocris) or 4 ng recombinant human ADAMTS4 (R&D Systems 4307-AD; 2 ng/side in 100 nl) were performed at ZT5 using bevelled stainless steel 33-gauge injectors, extending 1 mm beyond the tip of the guide. Plasma samples were collected from the tail vein 1 h after aCSF or ADAMTS4 injection at ZT6, after which mice were refed and returned to the holding room for daily body weight and food intake measurements in the week after injection. Insulin tolerance tests (ITTs) were started 1 h after aCSF/ADAMTS4 administration at ZT6 as described above.

### 2.11 Perfusion fixation

Mice were administered 50 ul pentobarbital (Dolethal, 200 mg/ml) by intraperitoneal injection to achieve deep anaesthesia. Mice were then trans-cardially perfused with 50 ml heparinised phosphate buffered saline (PBS) followed by 4% (w/v) paraformaldehyde (PFA; Fisher Scientific, Waltham, Massachusetts) in PBS (pH 7.4) or 4% PFA with 2.5% (v/v) glutaraldehyde (Sigma) in 0.1 M phosphate buffer (pH 7.4) at a flow rate of 5 ml/min. For BBB permeability assessments using Evans blue, mice were perfused with heparinised PBS followed by 4% PFA containing 1% Evans blue (w/v) at a flow rate of 7 ml/min. Following perfusion, brains were dissected and immersed in the appropriate fixative. PFA-fixed brains for immunofluorescent staining or cFos immunohistochemistry were transferred to 30% sucrose (w/v; Sigma) in PBS after 24–48 h in fixative.

### 2.12 Tissue processing

#### 2.12.1 Sectioning of PFA-fixed tissues

Following cryoprotection in 30% sucrose (w/v; Sigma) in PBS, PFA-fixed brains were embedded in Optimal Cutting Temperature (OCT; CellPath) and sectioned at 20–30 μm in the coronal plane from Bregma −1.58 to −2.30 mm [24] using a Leica SM2010R Sliding Microtome or Leica CM1950 Cryostat (Leica Biosystems). Except for sections obtained from mice with bilateral cannula, all sections were collected into cryoprotectant solution and processed as free-floating sections.

#### 2.12.2 Immunofluorescent staining

Sections were washed in PBS prior to heat mediated antigen retrieval in 20 mM sodium citrate buffer (pH 6.5; Fisher Scientific) for 20 min at 80 °C on a heat block. Where sections were immunolabelled for BrdU, sections were subjected to an additional antigen retrieval step in 2 N hydrochloric acid (Fisher Scientific) for 30 min in a water bath pre-heated to 37 °C followed by incubation with 0.1M sodium borate buffer (pH 8.5; Fisher Scientific) for 10 min at room temperature. Sections were then washed in PBS prior to blocking with 3–10% (v/v) normal donkey – or goat-serum (NDS/NGS; Abcam) in PBS with 0.3% (v/v) Triton X-100 (0.3% PBST) for 1 h at room temperature with agitation. Primary antibodies were prepared in blocking solution and incubated with sections for 1–2 nights at 4 °C with agitation. Sections were washed in 0.1% PBST prior to incubation with secondary antibodies diluted at 1:500 in 0.3% PBST for 2 h at room temperature. Antibodies targeting different antigen were multiplexed where possible using a sequential staining approach. Following staining, sections were mounted to slides under coverslips with VectaShield HardSet Antifade Mounting Medium with DAPI (Vector Laboratories), left to cure overnight at room temperature and stored at 4 °C until imaging.

Primary antibodies were goat anti-Sox10 (R&D Systems AF2864, 1:50), rabbit anti-PDGFRa (Cell Signalling Technologies 3164, 1:500), rat anti-BrdU (Abcam ab6326, 1:200), guinea pig anti-Bcas1 (Synaptic Systems 445 004, 1:500), rabbit anti-Bcas1 (Synaptic Systems 445 003, 1:1000), guinea pig anti-opalin (Synaptic Systems 457 005, 1:500), rat anti-MBP (Abcam ab7349, 1:500), chicken anti-GFP (Abcam ab13970, 1:1000), Wisteria floribunda agglutinin lectin (Vector Laboratories B-135, 1:500), rabbit anti-ADAMTS4 (Invitrogen PA1-1749A, 1:500), chicken anti-vimentin (Millipore AB5733, 1:1000), rabbit anti-Zo-1 (Invitrogen 61–7300, 1:250), rabbit anti-laminin (Sigma–Aldrich L9393, 1:500) and rat anti-MECA32 (NovusBio NB100-77668, 1:50).

Secondary antibodies were biotinylated goat anti-rabbit (Vector Laboratories BA-1000-15), goat anti-guinea pig 647 (ThermoFisher Scientific A21450), goat anti-rabbit 555 (ThermoFisher Scientific A21428), goat anti-rat 488 (ThermoFisher A11006), donkey anti-chicken 488 (Jackson ImmunoResearch 703-545-155), donkey anti-goat 647 (ThermoFisher Scientific A21447), donkey anti-rabbit 488 (ThermoFisher Scientific A21206), donkey anti-rabbit 555 (ThermoFisher Scientific A31572), donkey anti-rabbit 594 (ThermoFisher Scientific A21207), donkey anti-rat 488 (ThermoFisher Scientific A21208), Streptavidin 594 (ThermoFisher Scientific S11227) and Streptavidin 647 (ThermoFisher Scientific S21374).

#### 2.12.3 Confocal microscopy and analysis

Researchers were blinded to the experimental condition during imaging and analysis. Sections were imaged as z-stacks on a Leica SP8 confocal microscope at intervals of 2.0–3.3 μm using a 40x oil objective with tile scanning to obtain information from the entire depth and area of the region of interest (ROI). During image acquisition, microscope settings and z-stack size were identical within each experiment. Images were analysed using Fiji (ImageJ) software. For analysis, z-stacks were projected into a single image and the freehand tool was used to draw, define, and calculate the ROI area. Cell counts were performed using the Fiji manual cell counter tool. Where area was measured, the same threshold was applied to all images prior to measurement of the area or area fraction. ROI borders were determined using the Paxinos and Franklin Mouse Brain Atlas^44^.

#### 2.12.4 cFos immunohistochemistry

Sections were washed in PBS prior to incubation with 0.5% hydrogen peroxide (H_2_O_2_) in MilliQ water (MQ-H_2_O) for 15 min at room temperature. Following washing with PBS, sections were blocked in 5% NGS in 0.3% PBST for 1 h at room temperature and incubated with rabbit anti-cFos (Synaptic Systems 226 003) diluted 1:1000 in blocking solution for 2 nights at 4 °C with agitation. Sections were washed in 0.1% PBST prior to incubation with biotinylated goat anti-rabbit secondary antibody (Vector Laboratories BA-1000-15) diluted 1:500 in 0.3% PBST for 1 h at room temperature. ABC Reagent (Vector Laboratories PK-6100) was prepared as per the manufacturer’s instructions and, following washing with 0.1% PBST, was incubated with sections for 1 h at room temperature. Sections were washed in 0.1% PBST prior to incubation with 3′3′-Diaminobenzidine (DAB; Sigma D8001) solution (15 ml PBS, 500 μl 5 mg/ml DAB in MQ-H_2_O, 50 ul 1% H_2_O_2_) for 5–10 min at room temperature with agitation. Sections were washed in PBS, mounted to slides and dehydrated using increasing concentrations of ethanol (Sigma) prior to mounting under coverslips with Pertex mounting media (Fisher Scientific). Images were acquired using an Axioscan A1 Slide Scanner (Zeiss) using a 20x air objective. Images were analysed in a blinded manner by manual cell counting within ROIs in Zen Software (Zeiss).

### 2.13 RNAscope single molecule fluorescent *in situ* hybridisation

Perfusion-fixed brains were embedded in paraffin blocks and sectioned in the coronal plane at 5 μm on a Leica RM2235 microtome. Sections were dewaxed with xylene and 100% ethanol and washed prior to processing. Single molecule fluorescent *in situ* hybridisation was performed as previously described^11^ using RNAscope technology and a Leica Bond Rx instrument. Mouse *Bmp4* was labelled with a RNAscope 2.5 LS Probe (Mm-Bmp4-C2; ACD, 401308C2) and detected with Opal 570 (Akoya Biosciences, OPI-001006). Sections were imaged using a spinning disk Operetta SLS (PerkinElmer) in confocal mode with a sCMOS camera and 40x automated water-dispensing objective. All slides were imaged with identical gain and laser power settings and analysed in Harmony software (PerkinElmer).

### 2.14 Scanning electron microscopy (SEM)

Brains were post-fixed in 4% PFA with 2.5% (v/v) glutaraldehyde (Sigma) in 0.1 M phosphate buffer (pH 7.4) overnight before moving to 0.1M phosphate buffer. Brains were sectioned in the coronal plane on a Leica VT1000s vibratome to obtain 200 μm thick sections. Regions of interest were dissected out, washed 3 times in distilled water and incubated in 2% osmium tetroxide (Agar Scientific) with 1.5% potassium ferricyanide (Agar Scientific) for 2 h at 4 °C. Samples were then washed 3 times in distilled water and incubated with 2% uranyl acetate (Agar Scientific) overnight at 4 °C. The next morning, samples were washed 3 times in distilled water prior to dehydration through a series of washes in ethanol as follows: 25% for 15 min, 50% for 15 min, 75% for 15 min, 90% for 15 min, 100% for 15 min repeated twice. Samples were then embedded in Epoxy 812 resin (Agar Scientific) as follows: 12 h in a mixture of resin and 100% ethanol at a ratio of 1:3 respectively, 12 h in a mixture of resin and 100% ethanol at a ratio of 1:1 respectively, 12 h in a mixture of resin and 100% ethanol at a ratio of 3:1 respectively, 12 h in 100% resin repeated once. Samples were then cured at 70 °C for 48 h prior to sectioning to 100 nm on a Leica UC7 Ultramicrotome using a diamond knife (Diatome – Ultra 45). Once cut, sections were floated and collected on glow discharged silicon wafer chips to be imaged. Imaging was performed using a TESCAN Clara Scanning Electron Microscope at a magnification of 10,000X using a 2.5 kV electron beam and 300 picoamps of current. For statistical comparison of g-ratios between genotypes, individual animal means (*n* = 4 per genotype) were used as the unit of replication. The per-axon scatter plot is provided for visualisation of the distribution but was not used for inferential statistics.

G-ratio was quantified for at least 50 transverse axons per animal using Fiji (ImageJ) software by measuring the inner diameter of an axon and dividing by the outer diameter of the axon (axon + myelin sheath). Axons were excluded from analysis if they exhibited an awkward shape such that the diameter could not be defined. For axons demonstrating an elongated shape, the g-ratio was quantified from measurements taken perpendicular to the axis producing the longest diameter [25]. Quantification was only performed for axons displaying compact myelin sheaths. Axons were quantified in randomly selected fields of view (at least 5 per brain) from different regions within the ME to ensure inclusion of axons from the entire region of interest, including the ventral, dorsal, medial and lateral portions of the ME.

### 2.15 Whole brain clearing and 3D imaging

All brains were washed in phosphate-buffered saline (PBS, pH 7.4) for 3 × 30 min at room temperature. Whole-mount staining for GFP was performed according to the previously described iDISCO procedure [26] with slight modifications. Samples were dehydrated in increasing concentrations of methanol (20%, 40%, 60%, 80%, 100%) in H2O, and then washed in 100% methanol for 1 h and incubated overnight in 66% dichloromethane (DCM) and 33% methanol at room temperature. The next day, samples were washed twice in 100% methanol for 30 min, cooled down to 4 °C and bleached in chilled 5% H2O2 in methanol overnight at 4 °C. Samples were rehydrated in decreasing concentrations of methanol (80%, 60%, 40%, 20%) in PBS with 0.2% Triton X-100, 1 h each at room temperature, washed in PBS with 0.2% Triton X-100 for 2 × 1 h at room temperature, followed by incubation in permeabilization solution (PBS with 0.2% Triton X-100, 20% Dimethyl sulfoxide, DMSO) and 0.3 M glycine over three nights at 37 °C. This was followed by incubation in blocking solution (0.2% Triton X-100, 6% donkey serum, 10% DMSO and 0.02% sodium azide in PBS) over two nights at 37 °C. Subsequently, samples were incubated with anti-GFP antibody (1:2000, gfp-1020, Aves Labs) in antibody buffer (2% Tween-20, 5% DMSO, 3% donkey serum, 0.01 mg heparin/ml in PBS) at 37 °C for 7 days followed by a series of washes in washing solution (2% Tween-20 and 0.01 mg heparin/ml in PBS, 10 min, 20 min, 30 min, 1 h, 2 h and 2 × overnight). Samples were incubated with secondary antibody anti-chicken-Cy5 (1:1000, 703-175-155, Jackson ImmunoResearch) in antibody buffer over seven nights at 37 °C. This was followed by a series of washes in washing solution (10 min, 20 min, 30 min, 1 h, 2 h and three nights) and dehydration in increasing concentrations of methanol (20%, 40%, 60%, 80%, 100%) in H2O and incubation in 100% methanol overnight at room temperature. Samples were incubated in 66% DCM/33% methanol for 3 h at room temperature followed by 100% DCM for 2 × 15 min. Finally, the samples were transferred to dibenzyl ether (DBE).

#### 2.15.1 Light sheet microscopy

Brains were imaged using a Bruker Luxendo LCS SPIM microscope. Samples were mounted with ventral side down in a cuvette and imaged in ethyl cinnamate (ECi). Horizontal images were acquired at 4.4x magnification in a z-stack at 10 μm intervals. Autofluorescence images were capturesd at 560 ± 20 nm (excitation) and 650 ± 25 nm (emission) wavelength and the GFP signal was imaged at 630 ± 15 nm excitation wavelength and 680 ± 15 nm emission wavelength.

#### 2.15.2 Atlas registration

Region delineation of whole-brain samples were obtained by atlas segmentation. Atlas annotations were obtained by alignment to a digital LSFM-based mouse brain atlas [27]. The atlas utilizes the common coordinate framework version 3 (CCFv3) developed by the Allen’s Institute of Brain Science [28] and uses the same nomenclature. Registration between the atlas and the individual samples was performed by a global affine alignment followed by a local multi-resolution B-spline-based alignment, using the Elastix image registration toolbox [29]. The registration was based on the autofluorescence image volumes, which were pre-processed by down sampling to 20 μm isotropic resolution and contrast enhancement by contrast limited adaptive histogram equalization (CLAHE).

### 2.16 Quantitative polymerase chain reaction (qPCR)

Tissues for qPCR analysis were collected into RNALater solution (Ambion) and stored at −20 °C until processing. Total ribonucleic acid (RNA) was extracted using a Qiagen RNeasy Micro Kit (Qiagen) as per the manufacturer’s instructions in an RNase free environment. RNA quality and quantity was assessed spectrophotometrically using a NanoDrop ND-1000 (ThermoFisher Scientific) prior to the synthesis of single-stranded copy deoxyribonucleic acid (cDNA) using a High-Capacity cDNA Reverse Transcription Kit (Applied Biosystems) from 100 to 200 ng total RNA. qPCR was performed using SYBR Green chemistry on a QuantStudio 7.0 Flex Real-Time PCR System (Applied Biosystems) in 384 well plates. Relative gene expression was calculated using the 2ˆ(-ΔΔCt) method using a *Gapdh* as a housekeeping gene as the expression of this gene did not change between groups. All primers were obtained from Sigma–Aldrich, sequences can be found in Table 1.

**Table 1:** Primer sequences for SYBR Green qPCR.

Gene of interest	Forward primer	Reverse primer
*Acan*	GGCCACTGAAGGACAGGTC	ATTGGAGCGAAGGTTCTGGA
*Adamts4*	ATGGCCTCAATCCATCCCAG	AAGCAGGGTTGGAATCTTTGC
*Bcan*	TGCGCGTCAAGGTAAACGAA	GACCCCGGAATCATTGGGC
*Chst2*	CCGCTCGGGATGAAGGTATTT	CCACTTGTAGTCCAAGAGGTTGA
*Chst3*	GCCTTGGTCTACCGTGATGTC	GGAACTGAGTCAAGTGGTCCT
*Gapdh*	AGGTCGGTGTGAACGGATTTG	TGTAGACCATGTAGTTGAGGTCA
*Hapln1*	GGACCAGGACGCAGTGATTG	CGTGGTTTGGTGATTGGGTACT
*Mmp9*	CATTCGCGTGGATAAGGAGT	ACCTGGTTCACCTCATGGTC
Ptprz1	GGAGAAGAACAGAACATCGTCC	TCATTGCTCTGGTAATAGCCCA
*Tnc*	GCAGTGAAAAGCGGTGTCC	CTTCTCCGGTATAGCCCTCGT
*Tnr*	AGGCTGGTTTGGCAAGAACT	TCATCTCCGCTGTATTCACTGT
*Vcan*	GGAGGTCTACTTGGGGTGAG	GGGTGATGAAGTTTCTGCGAG

### 2.17 Analysis of HypoMap

Oligodendrocyte populations were isolated from HypoMap based on C7 cluster level labelling (C7-4 cluster is labelled as ‘Oligodendrocyte and precursor’. Visualisation of Adamts4 and other oligodendrocyte associated genes (Mog, Mbp, Bcas1, Bmp4, Tcf7l2, Pdgfra) was performed using Seurat v4 [30] and ggplot2. Average log-normalised expression of genes in each cluster at C66 level was used to create heatmaps of expression.

### 2.18 Statistical analysis

All data visualisation and statistical analysis was performed in Prism 10 Software (GraphPad). Details of statistical tests are found in figure legends. All data sets were tested for normality and equality of variance prior to significance testing. Normality testing was performed using a Shapiro–Wilk test. Equality of variance was tested using Bartlett’s test or Brown–Forsythe test. Values ± 2 standard deviations from the group mean were excluded from analysis, alternatively outliers were identified in GraphPad Prism using Grubb’s or ROUT’s test. All data are presented as mean ± standard error of the mean, n refers to the number of animals per group.

## 3 Results

### 3.1 Acute changes in glycaemia regulate oligodendrocyte lineage progression in the adult ME

**Figure 1:**
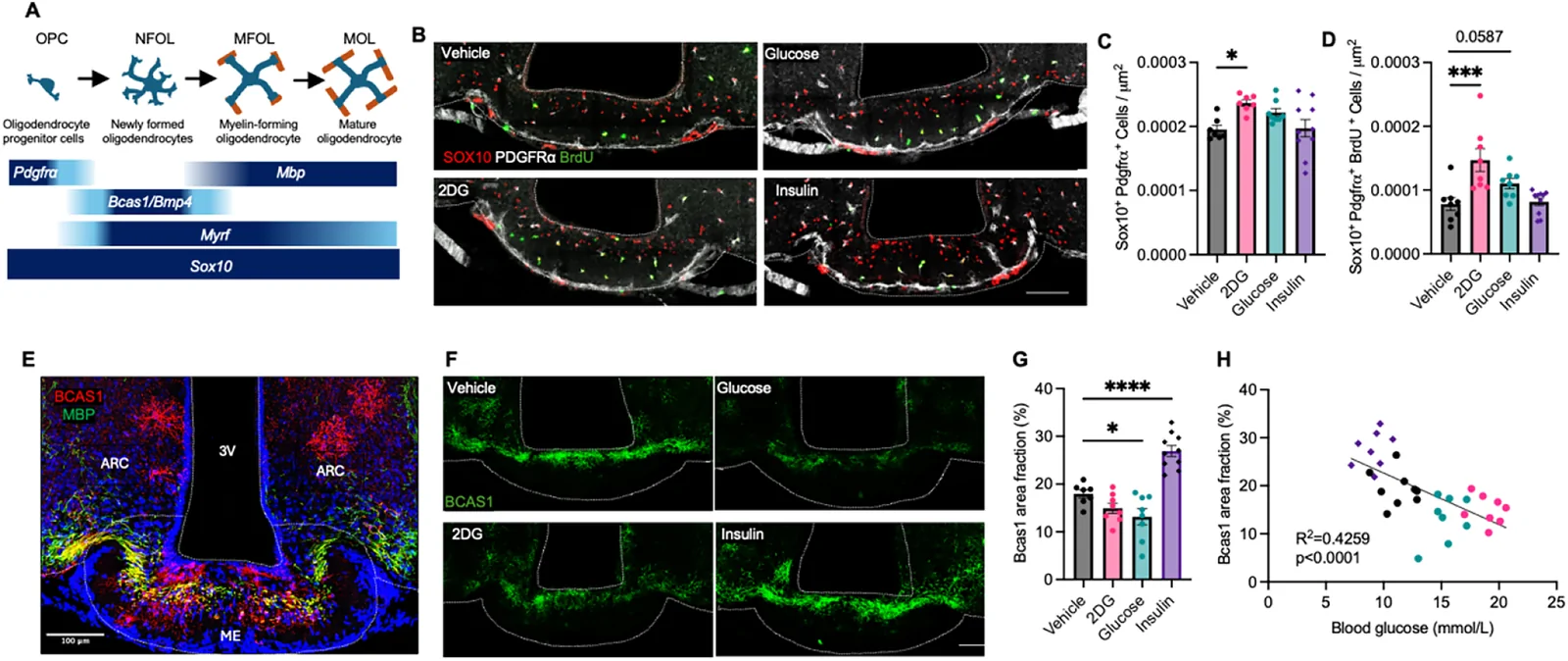
**Acute changes in glycaemia regulate oligodendrocyte lineage progression in the adult ME.** (**A**) Markers of oligodendrocyte lineage cell stages in the adult brain. **(B**) Representative images of pan-oligodendrocyte lineage marker SOX10, OPC marker PDGFRa and Bromodeoxyuridine (BrdU) in the mediobasal hypothalamus (MBH) of C57BL/6J mice 1 h after the intraperitoneal administration of vehicle (saline; 10 ml/kg; *n* = 7), 2-deoxy-d-glucose (2DG; 250 mg/kg; *n* = 8), glucose (2 g/kg; *n* = 8) or insulin (0.75 U/kg; *n* = 10) and the quantification of (**C**) OPCs (SOX10+/PDGFRa+) and (**D**) proliferating (BrdU+) OPCs in the ME. (**E**) Representative images of Breast carcinoma amplified sequence 1 (BCAS1), a marker of newly-formed (pre-myelinating) oligodendrocyte and myelin basic protein (MBP) immunolabelling in the MBH of a young adult male mouse. **(F)** Representative images of BCAS1 following vehicle, 2DG, glucose or insulin administration, (**G**) quantification of BCAS1 expression in the ME and (**H**) its correlation with blood glucose. Data analysed using one-way ANOVA (H) or Kruskal–Wallis (C,D) test with Dunn’s multiple comparisons test, ∗*p* < 0.05, ∗∗*p* < 0.01, ∗∗∗*p* < 0.001, ∗∗∗∗*p* < 0.0001. Correlations were analysed using simple linear regression, *n* = 8–10/group.

To determine how changes in blood glucose levels regulate ME oligodendrocyte lineage cells, we first characterised ME OPC proliferation in response to acute changes in blood glucose levels. C57BL/6J mice received intraperitoneal injections of vehicle (saline), glucose, 2-deoxy-d-glucose (2DG, a non-metabolizable glucose analogue) or insulin, 1 h prior to perfusion-fixation. As expected, glucose and 2DG administration resulted in hyperglycaemia (the latter reflecting the counter-regulatory response to glucoprivation) and insulin led to hypoglycaemia (Supplemental Fig. 1A). All mice also received bromodeoxyuridine (BrdU), a thymidine analogue that is incorporated into the DNA of actively dividing cells, to label proliferating cells as previously described [31]. Immunostaining against pan-oligodendrocyte marker SRY-box transcription factor 10 (SOX10) and OPC marker platelet derived growth factor receptor alpha (PDGFRα) was used to identify OPCs (SOX10+/PDGFRα+, Figure 1A). Remarkably within 1 h, 2DG significantly increased OPC proliferation (SOX10+/PDGFRα+/BrdU+) in the ME, leading to an increase in OPC density (Figure 1B–D). Glucose treatment resulted in a near-significant increase in OPC proliferation (SOX10+/PDGFRα+/BrdU+; Figure 1D). Thus, hyperglycaemia rapidly promotes ME OPC proliferation. In contrast, insulin treatment did not affect OPC proliferation or density (Figure 1B–D).

Next, to assess whether changes in glycaemia regulate OPC differentiation into oligodendrocytes in the ME, we quantified the expression of breast carcinoma amplified sequence 1 (BCAS1), a protein specifically expressed in newly-formed premyelinating oligodendrocytes (Fard et al., 2017; Figure 1E). 2DG administration had no effect on ME BCAS1 immunoreactivity. In contrast, BCAS1 expression significantly decreased following glucose administration and robustly increased in response to insulin administration (Figure 1F–G), suggesting that deviations from euglycaemia bidirectionally regulate OPC differentiation. In fact, blood glucose levels inversely correlate with ME BCAS1 expression (Figure 1H). Of note, despite the lack of change in OPC density and proliferation following insulin treatment, the density of oligodendrocyte lineage cells (SOX10+; representing OPCs and oligodendrocytes) significantly increased (Supplemental Fig. 1B) but expression of myelin basic protein (MBP) remained unchanged in the ME between vehicle and insulin treated groups (Supplemental Fig. 1C–D). Thus, insulin promotes the production of newly-formed premyelinating oligodendrocytes.

**Figure 2:**
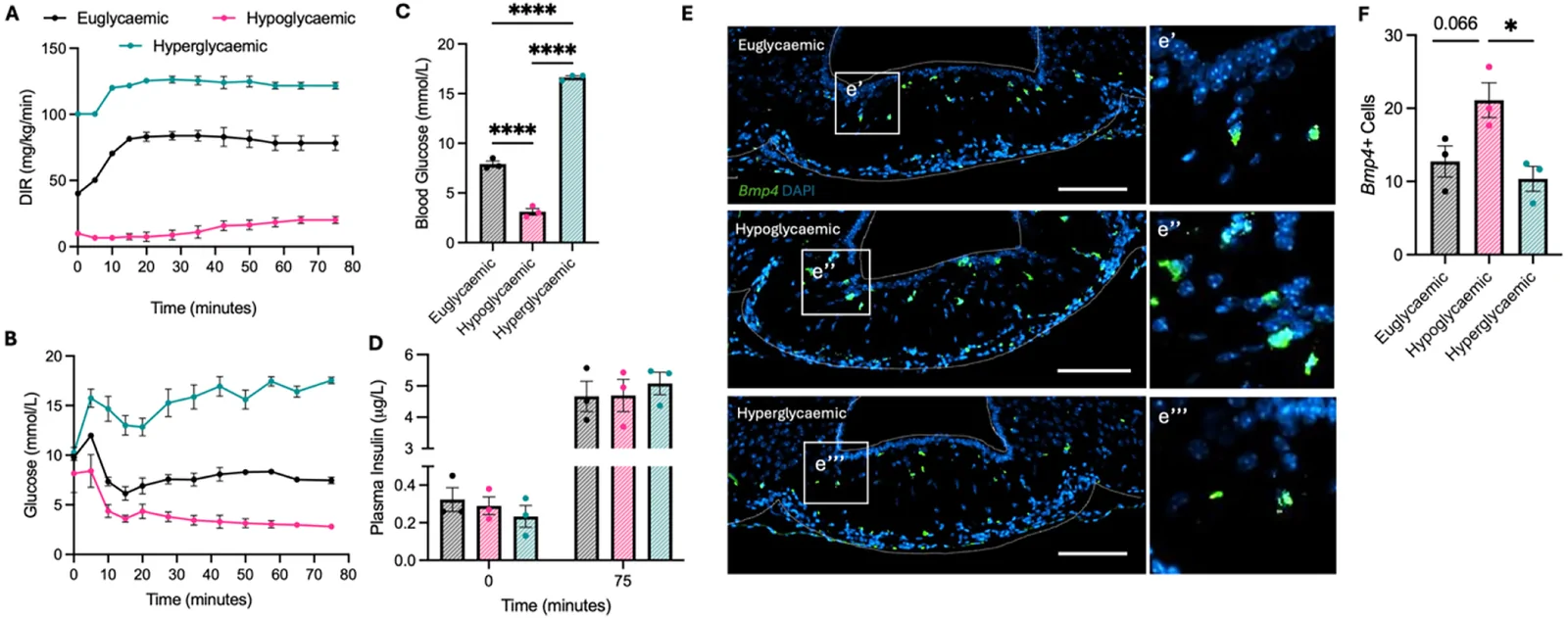
**Hypoglycaemia rapidly increases ME oligodendrocyte progenitor cell differentiation.** (**A**) Dextrose infusion rates (DIR; mg/kg/min) and (**B**) blood glucose levels (mmol/L) during a 75-minute hyperinsulinaemic-euglycaemic, - hypoglycaemic or hyperglycaemic clamp, (**C**) average blood glucose and (**D**) plasma insulin levels at 0 and 75 min. (**E**) Representative images of RNAscope single-molecule fluorescence *in situ* hybridization in the ME of C57BL/6J mice exposed to a 75-minute hyperinsulinaemic-euglycaemic, hypoglycaemic or hyperglycaemic clamp. Probes were used to label *Bone morphogenetic protein 4* (*Bmp4*) – expressed by newly differentiated oligodendrocytes. (**F**) Quantification of the number of cells expressing each marker, data presented as mean ± S.E.M. and analysed using one-way ANOVA with Tukey’s multiple comparisons test ∗*p* < 0.05, *n* = 3/group, scale bars represent 100 μm.

In insulin treated animals, either hyperinsulinemia or subsequent hypoglycaemia could drive the response of ME oligodendrocyte lineage cells. To tease these effects apart, we performed hyperinsulinaemic hypoglycaemic, euglycaemic and hyperglycaemic clamps in conscious, free moving mice (Figure 2A–C). Perfusion-fixed tissues were collected after 75-minutes for analysis of Bone morphogenetic protein 4 (Bmp4), a marker of newly differentiated oligodendrocytes [20,32], by single molecule fluorescent in situ hybridisation (sm-FISH, RNAscope technology [33]; Figure 2D). Consistent with our results indicating increased OPC differentiation into new oligodendrocytes following insulin administration, Bmp4 expression was significantly increased in the ME of mice following a 75-minute hyperinsulinaemic hypoglycaemic clamp (Figure 2E–F) confirming that hypoglycaemia rapidly increases the production of newly-formed premyelinating oligodendrocytes in the ME.

### 3.2 Adult oligodendrogenesis is required for the maintenance of glucose homeostasis

**Figure 3:**
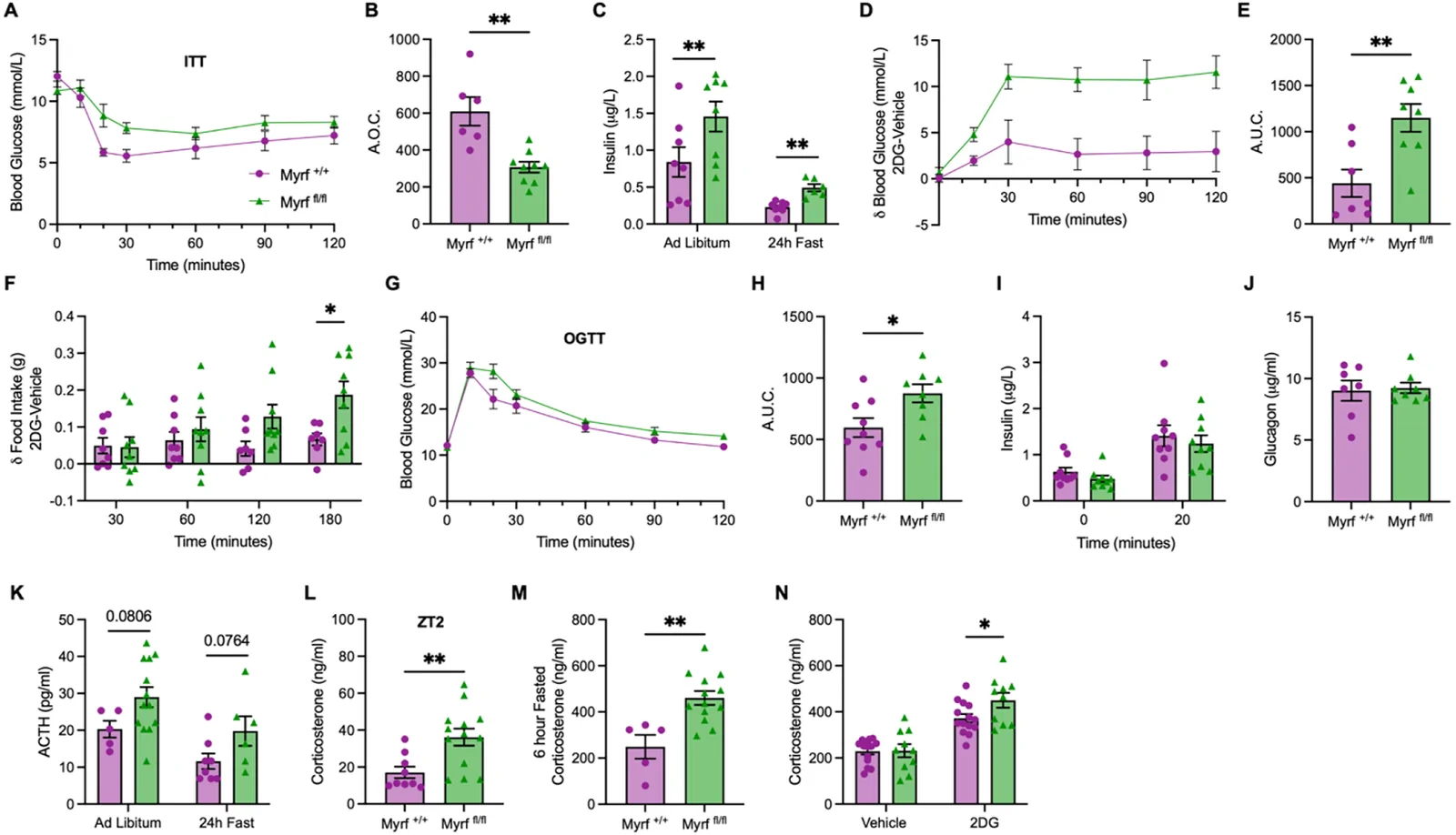
**Adult oligodendrogenesis is required for the maintenance of glucose homeostasis.** (**A**) Blood glucose levels in *Myrf*^*+/+*^*(*Pdgfra-CreERT2; Rosa26R-eYFP; Myrf^+/+^) and *Myrf*^*fl/fl*^ (Pdgfra-CreERT2; Rosa26R-eYFP; Myrf^f^ˡ^/f^ˡ) mice over 120 min following an intraperitoneal injection of insulin (0.75 U/kg, *n* = 6–9) and (**B**) the resulting area over the curve. (**C**) Plasma insulin levels in ad libitum fed and 24 h fasted *Myrf*^*+/+*^ and *Myrf*^*fl/fl*^ mice. (**D**) Blood glucose levels in *Myrf*^*+/+*^ and *Myrf*^*fl/fl*^ mice over 120 min following an intraperitoneal injection of 2-deoxy-d-glucose (2DG; 250 mg/kg, *n* = 8) compared to vehicle (saline; 10 ml/kg, *n* = 8) and (**E**) the resulting area under the curve. (**F**) Food intake over 180 min in *Myrf*^*+/+*^ and *Myrf*^*fl/fl*^ mice following an intraperitoneal injection of 2DG compared to vehicle. (**G**) Blood glucose levels in *Myrf*^*+/+*^ and *Myrf*^*fl/fl*^ mice over 120 min after an oral gavage of glucose (2 g/kg), (**H**) the resulting area under the curve and (**I**) plasma insulin levels at baseline and after 20 min. (**J**) Plasma glucagon levels in unchallenged *Myrf*^*+/+*^ and *Myrf*^*fl/fl*^ mice. (**K)** Plasma adrenocorticotrophic hormone levels in ad libitum fed and 24 h fasted *Myrf*^*+/+*^ and *Myrf*^*fl/fl*^ mice. Plasma corticosterone levels in *Myrf*^*+/+*^ and *Myrf*^*fl/fl*^ mice at (**L**) zeitgeber time (ZT) 2 and (**M**) after a 6 h fast. (**N**) Plasma corticosterone levels in *Myrf*^*+/+*^ and *Myrf*^*fl/fl*^ mice 30 min after vehicle or 2DG administration. All data presented as mean ± S.E.M. Data analysed using an unpaired student’s test (B, E, H, J, L, M) or two-way ANOVA with Sidak’s multiple comparisons test (A, C,D, F,G, I, K,N) ∗*p* < 0.05, ∗∗*p* < 0.01, *n* = 6–14/group.

**Figure 4:**
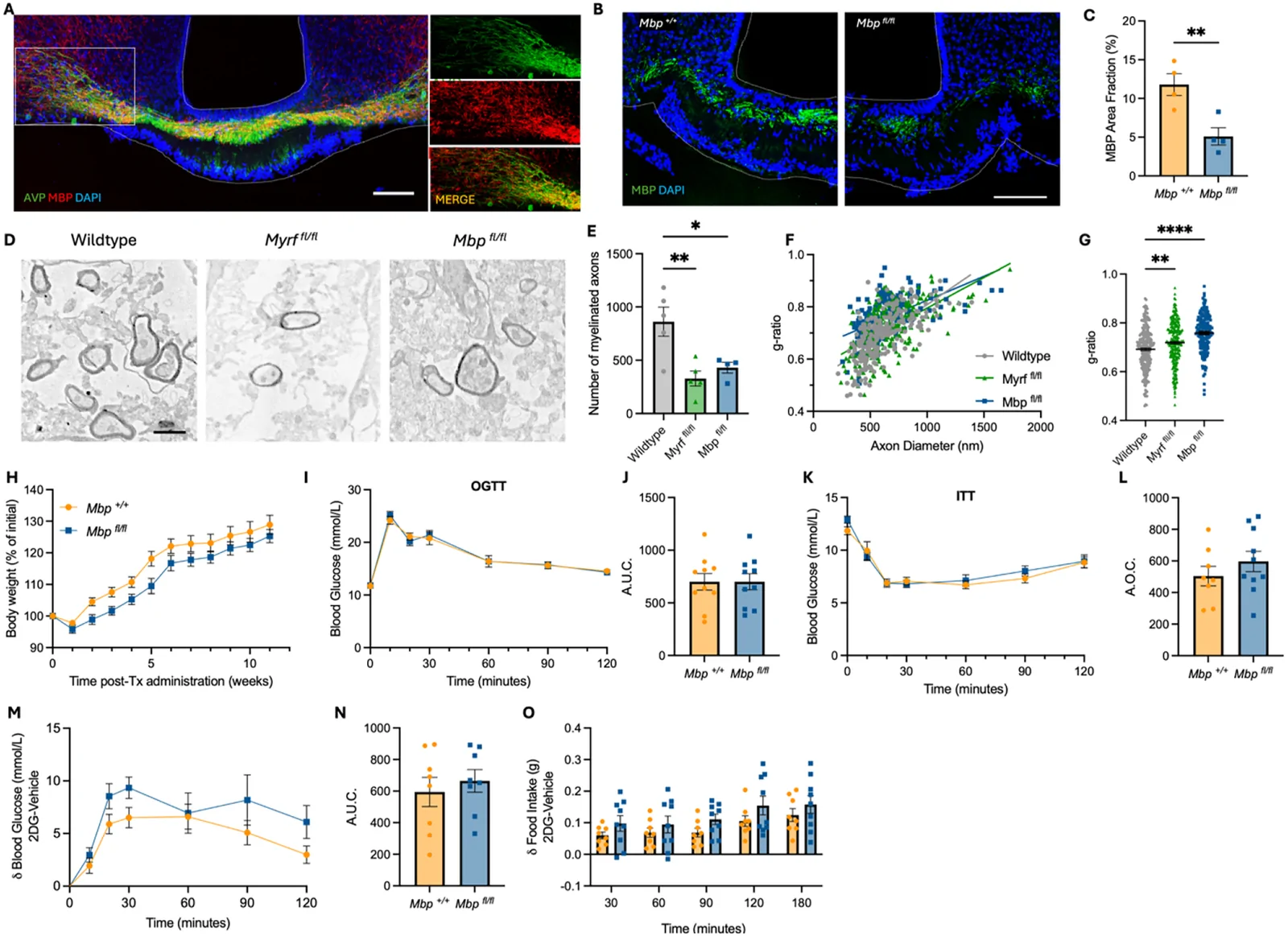
**Adult myelin generation is not required for the maintenance of glucose homeostasis**. (**A**) Representative images demonstrating myelination of arginine vasopressin (AVP) axons in the mediobasal hypothalamus. (**B**) Representative images of myelin basic protein (MBP) immunolabelling in the ME (ME) of MBP conditional knockout mice (*Mbp*^*fl/fl*^*,* Pdgfra-CreERT2; Rosa26R-eYFP; Mybp^fl/fl^) and controls (*Mbp*^*+/+*^, Pdgfra-CreERT2; Rosa26R-eYFP; Mybp^+/+^*n* = 4) six weeks after tamoxifen administration at postnatal day 60 and (**C**) associated quantification. (**D**) Representative images of myelin ultrastructure and integrity in the ME of wildtype, myelin regulatory factor conditional knockout (*Myrf*^*fl/fl*^, *n* = 4) and *Mbp*^*fl/fl*^ (*n* = 4) mice by serial transmission electron microscopy and associated quantifications of (**E**) the number of myelinated axons and (**F-G**) g ratio. (**H**) Body weight change over 12 weeks after tamoxifen administration to *Mbp*^*+/+*^ and *Mbpfl/fl* mice (*n* = 10). (**I**) Blood glucose over 120 min after an oral gavage of glucose (2 g/kg) in *Mbp*^*+/+*^ and *Mbpfl/fl* mice and (**J**) the associated area under the curve (*n* = 10). (**K**) Blood glucose over 120 min following an intraperitoneal injection of insulin (0.75 U/kg) and (**L**) the associated area over the curve (*n* = 10). (**M**) Blood glucose levels in *Mbp*^*+/+*^ and *Mbpfl/fl* mice over 120 min following an intraperitoneal injection of 2-deoxy-d-glucose (2DG; 250 mg/kg, *n* = 8) compared to vehicle (saline; 10 ml/kg, *n* = 8) and (**N**) the resulting area under the curve. (**O**) Food intake over 180 min in *Mbp*^*+/+*^ and *Mbpfl/fl* mice following an intraperitoneal injection of 2DG compared to vehicle. All data presented as mean ± S.E.M., scale bars represent 100 μm. Data analysed using an unpaired student’s test (C, J, L, N), one-way ANOVA with Dunnett’s multiple comparisons test (E, G) or two-way ANOVA with Sidak’s multiple comparisons test (H, I, K, M, O), ∗*p* < 0.05, ∗∗*p* < 0.01, ∗∗∗∗*p* < 0.0001.

The maintenance of glucose homeostasis relies on brain glucose sensing mechanisms [34]. Having identified ME oligodendrogenesis as a pathway responsive to changes in blood glucose levels, we then tested the role of adult oligodendrogenesis in glycemic control. We used Pdgfra-CreERT2; Rosa26R-eYFP; Myrffl/fl (Myrffl/fl) mice, where conditional deletion of myelin regulatory factor (Myrf) in OPCs blunts OPC differentiation following tamoxifen administration at postnatal day 60 (P60) [21] effectively blocking new oligodendrocyte production [21]. Compared to wild-type littermates (Myrf+/+), Myrffl/fl mice became resistant to the hypoglycaemic action of insulin within six weeks of tamoxifen administration (Figure 3A–B). Consistent with the development of insulin resistance, plasma insulin levels in both the fasted and ad libitum fed state were significantly increased in Myrffl/fl mice (Figure 3C). When challenged with 2DG, Myrffl/fl mice mounted an exacerbated hyperglycaemic (Figure 3D–E, Supplemental Fig. 2A–B) and hyperphagic (Figure 3F, Supplemental Fig. 2C) response, indicating an impaired counterregulatory response to neuroglycopenia. Finally, Myrffl/fl mice displayed glucose intolerance when challenged with an oral gavage of glucose (Figure 3G–H). However, glucose-induced hyperinsulinemia did not differ between groups (Figure 3I) nor did plasma glucagon levels in unchallenged mice (Figure 3J) suggesting normal pancreatic function following Myrf deletion (see Figure 4).

The glucose counterregulatory response which orchestrates the recovery from hypoglycaemia involves activation of the hypothalamic pituitary adrenal (HPA) axis through the release of corticotropin-releasing hormone (CRH) in the ME from the hypothalamic paraventricular nucleus (PVH). CRH reaches the anterior pituitary through the hypophyseal portal circulation to stimulate ACTH synthesis, which in turn stimulates corticosterone secretion from the adrenal cortex. Thus, we next examined the activity of the HPA axis during blockade of adult oligodendrogenesis. In Myrffl/fl mice, plasma levels of adrenocorticotrophic hormone (ACTH) were increased to near statistical significance under ad libitum feeding conditions at zeitgeber time (ZT) 2 or following a 24 h fast (Figure 3K). Consistently, plasma corticosterone levels were significantly elevated in Myrffl/fl mice at ZT2 (Figure 3L) and showed a similar trend after a 6-hour fast (Figure 3M), although this comparison did not reach statistical significance. Together with the significant elevation at ZT2 and the exacerbated corticosterone response to 2DG (Figure 3N, see below), these data collectively support a state of heightened HPA axis reactivity upon blockade of adult oligodendrogenesis. Finally, we assessed the ability of Myrffl/fl mice to mount an appropriate neuroendocrine response to glucoprivation, which involves increases in circulating levels of corticosterone and glucagon to prevent a further decline in plasma glucose levels and restore euglycaemia [35]. Consistent with a heightened sensitivity to glucoprivation, Myrffl/fl mice displayed an exacerbated hypercorticosteronaemic, but not hyperglucagonemic response to 2DG (Figure 3N, Supplemental Fig. 2D). Collectively, these data support a role for adult oligodendrogenesis in the regulation of the HPA axis and the activation of counterregulatory mechanisms in response to hypoglycaemia.

### 3.3 Adult myelin generation is not required for the maintenance of glucose homeostasis

In the ME, myelin rapidly turns over [21], and genetic block of oligodendrocyte differentiation in adult Myrffl/fl mice leads to rapid and significant reduction in ME myelin basic protein (MBP) expression, a proxy for myelination [21]. Since ME myelin ensheaths axons of magnocellular arginine vasopressin (AVP) neurons (Pastor et al., 1991; Fig. 4A), which have been implicated in the regulation of glucose homeostasis [36], demyelination in the ME of *Myrf*^*fl/fl*^ mice could mediate their neuroendocrine and glycaemic phenotype.

To test this hypothesis, we first assessed the integrity of the AVP neuroendocrine axis in *Myrf*^*fl/fl*^ mice focussing on its role in osmoregulation. ME AVP immunolabelling was unchanged in *Myrf*^*fl/fl*^ mice (Supplemental Fig. 3A–B) and ad libitum water intake was comparable to controls (Supplemental Fig. 3C). Furthermore, Myrffl/fl mice exhibited the expected increase in plasma osmolarity during a dehydration challenge (Supplemental Fig. 3D), suggesting intact osmoregulation. Extending on these findings, we visualised new myelin production in the adult brain between P60 and P90 in Pdgfrα-CreERT2;taumGFP mice dosed with tamoxifen at P60 as previously described [21]. We used tissue clearing and light-sheet 3D imaging of the tau-mGFP signal, which in this model localises to adult-generated myelin [21]. Strikingly, new myelin formation was restricted to the ME in the hypothalamus and absent from the paraventricular nucleus of the hypothalamus and the supraoptic nucleus, where magnocellular AVP cell bodies reside (Fig. 4E–F, Suppl. Video 1). This suggests that, in this timeframe, myelin plasticity on AVP axons is restricted to their terminal segments at the level of the ME.

To directly evaluate the role of adult myelin generation in the regulation of glucose homeostasis, we used *Pdgfra-CreERT2*;*Rosa26R-eYFP*;*Mbp*^*fl/fl*^ (*Mbpfl/fl*) mice in which tamoxifen-induced deletion of the *Mbp* gene promoter from OPCs blocks *Mbp* transcription, MBP protein accumulation and myelin compaction in newly-formed myelin sheaths. This allows us to specifically assess the contribution of adult-formed myelin to the regulation of glucose homeostasis. Deletion of *Mbp* in OPCs led to a robust (∼50%) reduction in ME MBP expression 6 weeks after tamoxifen administration at P60 in *Mbpfl/fl* mice (Fig. 4B–C) and was associated with a decrease in the total number of oligodendrocyte lineage cells (SOX10+) and a near-significant reduction in oligodendrocyte (*Sox10*+/PDGFRα−) density in the ME (Supplemental Figure 3G–H). We then used serial electron microscopy (SEM) to assess myelin ultrastructure in the ME of both *Myrffl/fl* and *Mbpfl/fl* mice compared to wild-type controls (Fig. 4D). As expected, both deletion of *Myrf* and *Mbp* from OPCs significantly reduced the number of myelinated axons in the ME (Fig. 4E) and quantification of the g-ratio on axons displaying compact myelin sheaths demonstrated a significant increase in the g-ratio in both *Myrffl/fl* and *Mbpfl/fl* mice compared to wild-type controls indicating thinner myelin sheaths (Fig. 4F–G). Despite changes in ME myelin density and structure and the number of cells in the oligodendrocyte lineage, the density of newly formed oligodendrocytes (*Sox10*+/PDGFRα−/YFP+; Supplemental Fig. 3G, 3I) did not change in the ME following conditional deletion of *Mbp* in OPCs. Consistently, BCAS1 expression was comparable between *Mbpfl/fl* mice and littermate controls (Supplemental Fig. 3J–K) indicating that deletion of *Mbp* from adult OPCs does not impact ME OPC differentiation or new oligodendrocyte production, and specifically impairs compactness of adult-formed myelin.

Phenotypically, deletion of *Mbp* in OPCs at P60 produced a trend towards reduced weight gain (genotype effect p=0.0803, interaction (time∗genotype) p=0.0725; Fig. 4H). However, unlike what we observed in *Myrffl/fl* mice, glucose tolerance, insulin sensitivity and the response to 2DG-induced glucoprivation did not differ between *Mbpfl/fl* mice and wild-type littermates (Fig. 4I–O, Supplemental Fig. 3L–N). Mbpᶠˡ^/^ᶠˡ mice showed a modest increase in cumulative ad libitum food intake compared to wild-type littermates (Supplemental Fig. 3N) that was not accompanied by changes in body weight (Fig. 4H), glucose tolerance (Fig. 4I–J), insulin sensitivity (Fig. 4K–L) or the glucoprivic response to 2DG (Fig. 4M–O). This dissociation indicates that the mild hyperphagia of Mbpᶠˡ^/^ᶠˡ mice is compensated at the level of energy expenditure or nutrient handling rather than reflecting disrupted counter-regulatory control. Thus, the adult generation of new compact myelin is dispensable for the regulation of glucose homeostasis in adult mice.

### 3.4 Blockade of adult oligodendrogenesis remodels the blood-hypothalamus barrier

The ME contributes to energy and glucose homeostasis by orchestrating bidirectional communications between central and the peripheral endocrine systems. Specialised glial populations of the ME, including tanycytes and fenestrated capillaries, are dynamically remodelled in response to metabolic challenges such as energy or glucose deficit [37,6]. However, whether ME oligodendrocyte lineage cells contribute to these adaptations is unknown. Hence, we next examined ME barrier properties in *Myrf*^*fl/fl*^ mice.

**Figure 5:**
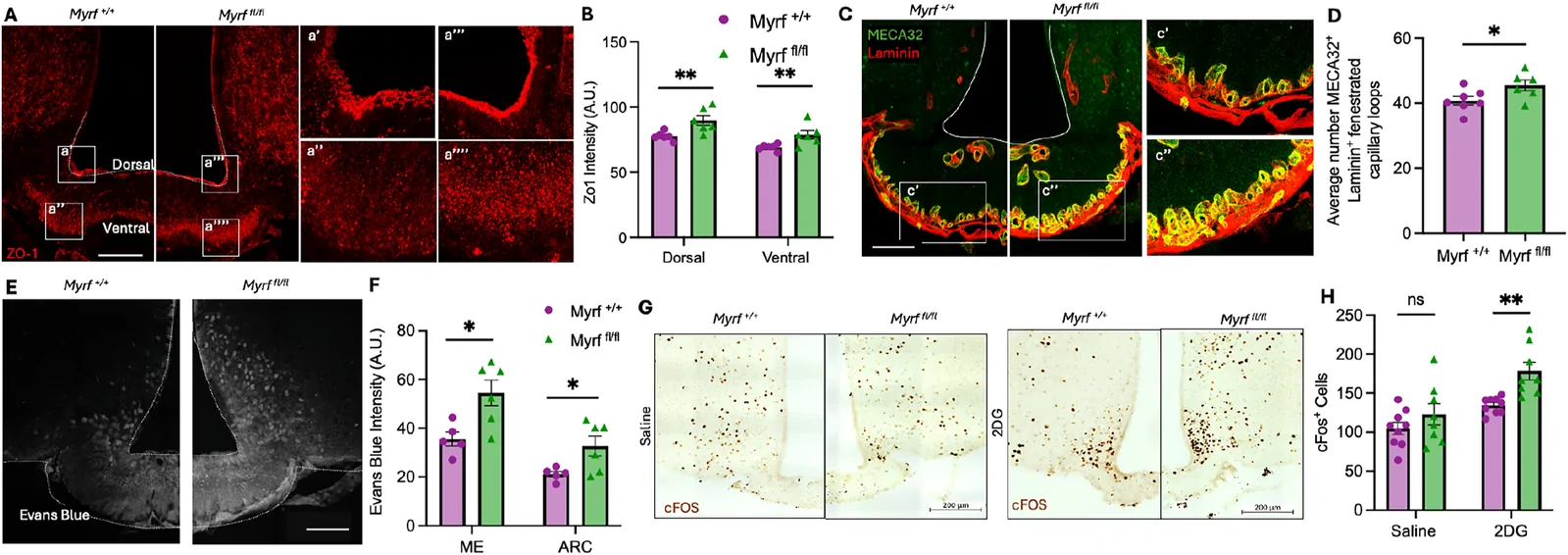
**Blockade of adult oligodendrogenesis remodels the blood-hypothalamus barrier** (**A**) Representative images of Zona occludens 1 (ZO-1) immunolabelling in the mediobasal hypothalamus (MBH) of myelin regulatory factor (MYRF) conditional knockout mice (*Myrf*^*fl/fl*^, *n* = 6) and controls (*Myrf*^*+/+*^, *n* = 6–7) and (**B**) associated quantification. (**C**) Representative images of laminin and pan-endothelial cell marker MECA-32 in the MBH of *Myrf*^*fl/fl*^ and *Myrf*^*+/+*^ mice and (**D**) associated quantification. (**E**) Evans blue permeation of the mediobasal hypothalamus (MBH) in *Myrffl/fl* and *Myrf*^*+/+*^ mice following an intravascular bolus at zeitgeber time 2 and (**F)** associated quantification. (**G**) Representative images of cFOS immunohistochemistry in the MBH 90 min after an intraperitoneal injection of saline (10 ml/kg, *n* = 8) or 2-deoxy-d-glucose (2DG; 250 mg/kg, *n* = 8) to *Myrf*^*+/+*^ and *Myrffl/fl* mice and (**H**) associated quantification of cFOS + cells in the arcuate nucleus (ARH). Data presented as mean ± S.E.M., scale bars represent 100 μm. Data analysed using either an unpaired student’s-t-test (B,D,F), or 2-way ANOVA with Sidak’s multiple comparisons test (H), ∗*p* < 0.05, ∗∗*p* < 0.01.

ME-ARH tanycytic morphology and density were unchanged in *Myrf*^*fl/fl*^ mice (Supplemental Fig. 4A–B), but the expression of the tight-junction protein Zona occludens 1 (ZO-1) was increased in both the dorsal and ventral ME (Figure 5A–B), suggesting decreased paracellular permeability and remodelling of local barriers. Further, the number of MECA32+/Laminin + capillary loops was increased in the ME of *Myrf*^*fl/fl*^ mice (Figure 5C–D) indicating increased blood vessel fenestration and BHB permeability. Consistently, Evans Blue infiltration into the ME-ARH following an intra-vascular bolus was greater in *Myrf*^*fl/fl*^ mice compared to controls (Figure 5E–F), indicating reduced ME-ARH barrier function and increased access of circulating signals to the hypothalamus. We hypothesised that reduced blood-hypothalamus barrier function in *Myrf*^*fl/fl*^ mice might contribute to their glycemic phenotype by increasing the exposure of hypothalamic glucose-sensing neurons to hypoglycaemic or neuroglucopenic challenges. To test this, we measured cFOS expression in the ARH of *Myrf*^*fl/fl*^ mice and littermate controls 90 min after ip administration of saline or 2DG (Figure 5G). Consistent with the exacerbated hyperphagic and hyperglycaemic response to 2DG in *Myrf*^*fl/fl*^ mice (Figure 3D–F), the number of cFOS + cells in the ARH was significantly higher in *Myrf*^*fl/fl*^ mice compared to controls following treatment with 2DG (Figure 5H).

### 3.5 Adult oligodendrogenesis is required for the maintenance of hypothalamic perineuronal nets and ADAMTS4 expression

Hypothalamic perineuronal nets (PNNs) are emerging as putative structural components of the blood-hypothalamus barrier [38,39]. Since OPCs and newly-formed oligodendrocytes largely contribute to the synthesis of PNN core proteins and enzymes mediating PNN remodelling [20], we examined the possibility that altered hypothalamic PNN deposition in Myrffl/fl mice might contribute to changes in blood-hypothalamus barrier permeability in this model.

**Figure 6:**
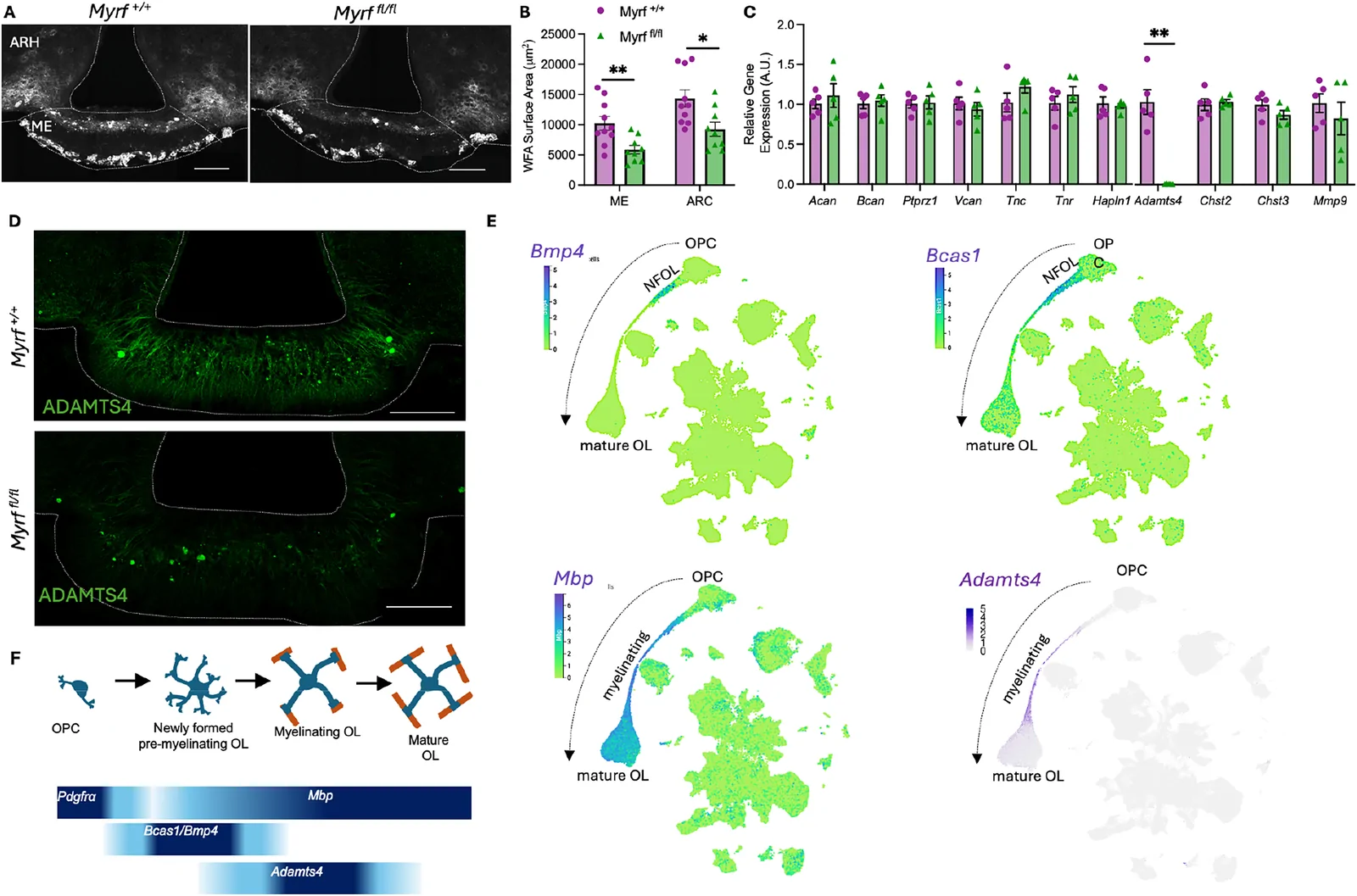
**Adult oligodendrogenesis is required for the maintenance of hypothalamic perineuronal nets and ADAMTS4 expression**. (A) Representative images of Wisteria floribunda agglutinin (WFA) lectin labelling in the ME and ARH of myelin regulatory factor (Myrf) conditional knockout mice (*Myrf*^*fl/fl*^, *n* = 9) and controls (Myrf+/+, *n* = 10) six week after tamoxifen administration at postnatal day 60 and (B) associated quantification. (C) Gene expression analysis of transcripts relating to perineuronal net (PNN) structural components and modifying enzymes in the mediobasal hypothalamus of Myrf+/+ and *Myrf*^*fl/fl*^ mice (*n* = 5). (D) Representative images of ADAMTS4 labelling in the ME-ARH of Myrf+/+ and *Myrf*^*fl/fl*^ mice. (E) Log-normalized expression of Bmp4, Bcas1 and Mbp. (F) Log-normalized expression of Adamts4 in the hypothalamic cell populations from HypoMap data [32]. Data presented as mean ± S.E.M. and analysed by unpaired student’s (B) - or Welch’s (C) – t test, ∗*p* < 0.05, ∗∗*p* < 0.01, scale bars represent 100 μm.

Visualisation of chondroitin sulphate (CS)-GAGs using Wisteria floribunda agglutinin (WFA) lectin demonstrated a significant reduction in the density of WFA + PNNs in the ME and ARH of Myrffl/fl mice (Figure 6A–B), indicating that active oligodendrogenesis is required for PNN maintenance in the ME. To further evaluate how genetic block of new oligodendrocyte production might lead to these changes, we measured the relative gene expression of PNN structural components expressed by ME oligodendrocyte lineage cells including CSPGs, glycoproteins, link proteins and enzymes mediating PNN post-translational modifications. None of these transcripts were differentially expressed in Myrffl/fl mice compared to littermate controls, except for Adamts4 (A disintegrin and metalloproteinase with thrombospondin motifs 4) which was strikingly absent from the ME of Myrffl/fl mice (Figure 6C). Lack of Adamts4 transcript in the ME of Myrffl/fl mice was associated with a robust decrease in ME ADAMTS4 protein expression (Figure 6D). Strikingly, transcriptomic data indicate that brain Adamts4 is expressed exclusively by oligodendrocytes [40,41] and specifically enriched in oligodendrocytes undergoing active myelination [42]. To further confirm the molecular identity of the oligodendrocyte subset expressing Adamts4 in the ME, we analysed the expression of Adamts4 in the HypoMap [32], a publicly available transcriptomic reference map of the murine hypothalamus. This confirmed that hypothalamic Adamts4 expression is restricted to oligodendrocyte lineage cells (Figure 6E, Supplemental Fig. 5), and enriched in newly formed actively myelinating oligodendrocytes (Figure 6E, Supplemental Fig. 5).

### 3.6 ADAMTS4 regulates the blood-hypothalamus barrier, hypothalamic glucose sensing and glucose homeostasis

**Figure 7:**
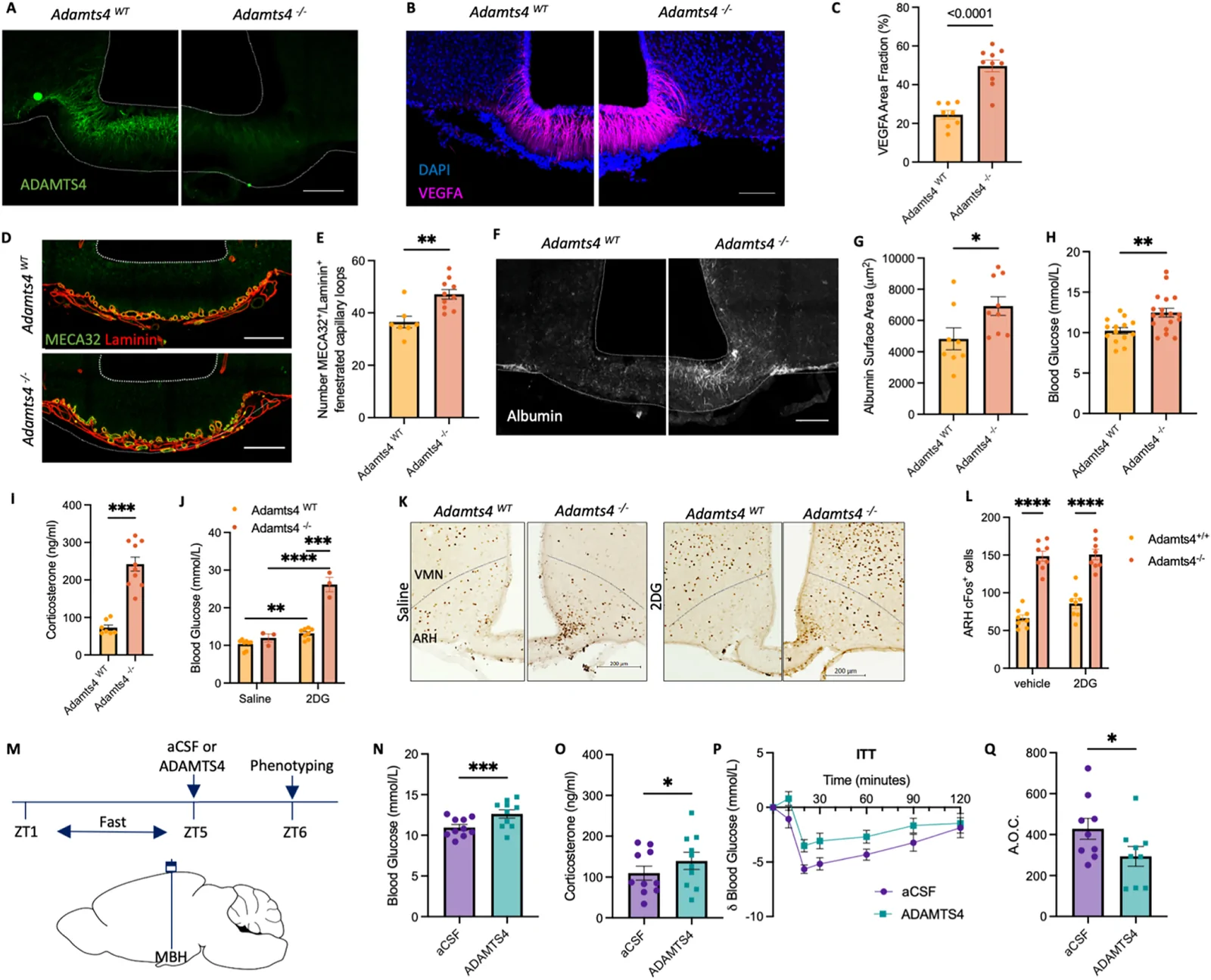
**ADAMTS4 regulates the blood-hypothalamus barrier, hypothalamic glucose sensing and glucose homeostasis.**(**A**) ADAMTS4 expression in the MBH of ADAMTS4 knockout (*Adamts4*^*−/−*^, *n* = 10) mice compared to controls (*Adamts4*^*WT*^, *n* = 8). Representative images of (**B**) VEGFA, (**D**) MECA32+/laminin + fenestrated capillary loops and (**F**) albumin immunolabelling in *Adamts4*^*−/−*^ and *Adamts4*^*WT*^ mice and associated quantifications (**C, E, G**). (**H**) Blood glucose and (**I**) plasma corticosterone levels in *Adamts4*^*−/−*^ and *Adamts4*^*WT*^ mice at ZT2. (**J**) Blood glucose, and (**K,L**) MBH cfos immunolabelling in *Adamts4*^*−/−*^ and *Adamts4*^*WT*^ mice 90 min following an ip injection of saline (*n* = 7–8) or 2DG (*n* = 3–7). (**N**) Blood glucose, (**O**) plasma corticosterone and (**P-Q**) insulin sensitivity in C57BL/6J mice 1 h after intra-hypothalamic aCSF or ADAMTS4 administration (**M**). Data presented as mean ± S.E.M. and analysed by unpaired student’s t-test (C, E, G, H, I, N, O, Q) or Mann–Whitney test (J, L), ∗*p* < 0.05, ∗∗*p* < 0.01, ∗∗∗*p* < 0.001, ∗∗∗∗*p* < 0.0001. Scale bars represent 100 μm.

ADAMTS4 is a metallopeptidase important for vascular remodelling [43,44], raising the possibility that lack of ADAMTS4 in Myrffl/fl mice might contribute to blood-hypothalamic barrier changes observed in this model. To test this, we examined hypothalamic tissues from Adamts4 knockout (Adamts4−/−) mice. We confirmed absence of ADAMTS4 expression in the ME (Figure 7A). ME MBP expression was unaffected by Adamts4 knockout (Supplemental Fig. 6A–B) while BCAS1 expression was increased (Supplemental Fig. 6C–D). Thus, Adamts4 deletion does not impair new oligodendrocyte generation and myelination in the ME. Consistent with the anti-angiogenic role of ADAMTS4, we measured a significant increase in ME VEGFA expression in Adamts4 knockout mice (Figure 7B,C) alongside an increase in the number of MECA32+/Laminin + fenestrated capillary loops in the ventral ME (Figure 7D–E), supporting a role for ADAMTS4 in ME vascular remodelling. We used albumin immunostaining to assess permeability of the blood-hypothalamus barrier in this model [45] and measured an increase in albumin diffusion within the ME and ARH in Adamts4−/− mice (Figure 7F–G). Thus, Adamts4 deletion is sufficient to increase vessel fenestration and vascular permeability of the blood-hypothalamus barrier, mimicking alterations in the blood-hypothalamus barrier observed in Myrffl/fl mice. Further, blood glucose and plasma corticosterone levels were significantly increased in Adamts4−/− mice compared to controls (Figure 7H–I). Following a 2DG injection, Adamts4−/− mice exhibited an aberrant hyperglycaemic response, associated with hyperactivation of ARH neurons (Figure 7J–L), indicating that ADAMTS4 is required for the maintenance of glucose homeostasis.

To test whether gain-of-function of ADAMTS4 would produce reciprocal changes in ME structure and glycemic control, we performed bilateral injections of mouse recombinant ADAMTS4 directly into the MBH of adult C57BL/6J mice (Figure 7M). The density of fenestrated capillary loops in the ME of ADAMTS4 injected animals was unchanged after 1 h (Supplemental Fig. 6E–F). Moreover, against our prediction, MBH ADAMTS4 administration also increased blood glucose levels (Figure 7N) and plasma corticosterone (Figure 7O) within 1 h following injection, and significantly decreased insulin sensitivity (Figure 7P–Q), suggesting a bimodal role of ADAMTS4 in the regulation of blood glucose and corticosterone levels.

### 3.7 ME ADAMTS4 expression is downregulated during hypoglycaemia

Last, we aimed to resolve the relevance of ADAMTS4-mediated blood-hypothalamus barrier remodelling in response to glycaemic challenges. Because deletion of *Myrf* broadly depletes the ME from oligodendrocytes across all stages of differentiation and maturation, this might confound the identification of the specific functions of distinct stages of the oligodendrocyte lineage in response to changes in blood glucose levels. In fact, hypoglycaemia promotes OPC differentiation and the production of premyelinating oligodendrocytes without changing MBP expression (Figure 1), suggesting a specific increase in premyelinating oligodendrocytes. Since ADAMTS4 is expressed in myelinating oligodendrocytes, the effect of hypoglycaemia on ME ADAMTS4 expression is unclear.

**Figure 8:**
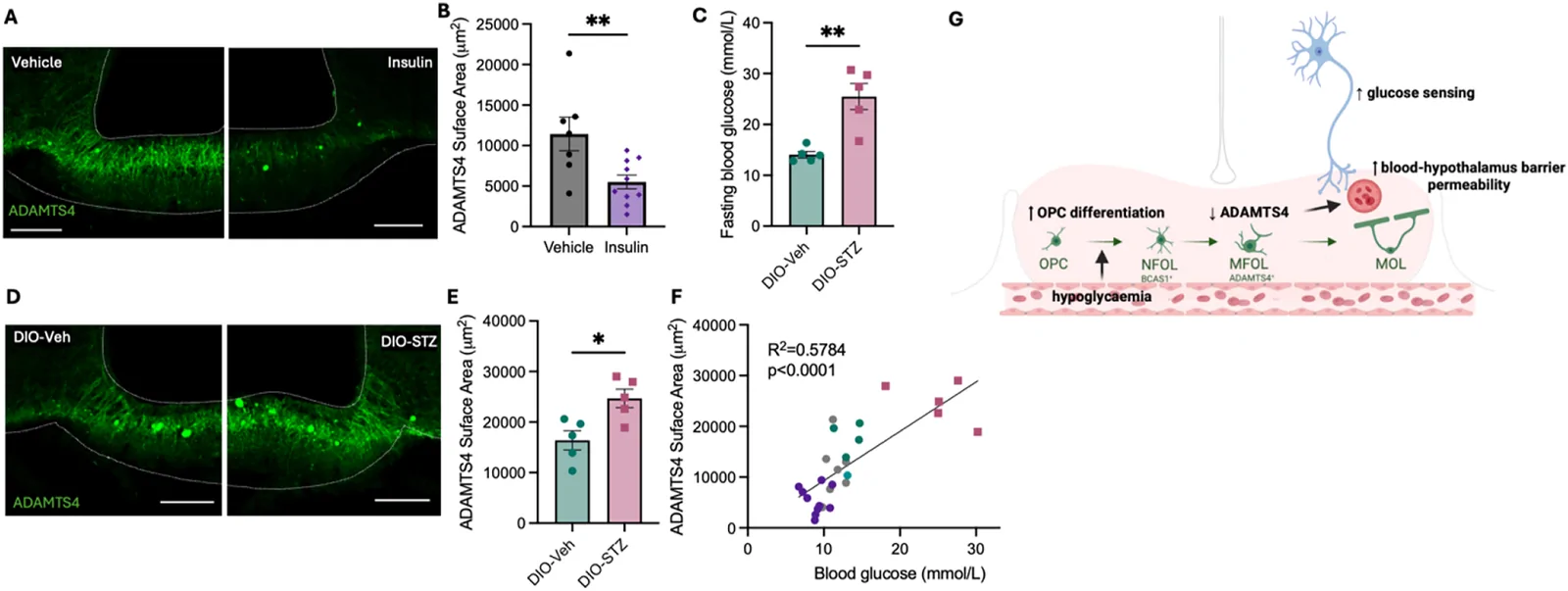
**ME ADAMTS4 expression is regulated by glycaemia**. Representative images of A disintegrin and metalloproteinase with thrombospondin motifs 4 (ADAMTS4), immunolabelling in the mediobasal hypothalamus (MBH) (**A**) 1 h after intraperitoneal administration of vehicle (saline; 10 ml/kg, *n* = 7) or insulin (0.75 U/kg, *n* = 10) and (**B**) associated quantification. (**C**) Blood glucose levels in mice fasted for 6 h in diet-induced-obese (DIO, *n* = 5) mice four weeks after administration of vehicle (DIO-Veh, *n* = 5) or streptozotocin (DIO-STZ, *n* = 5). (**D**) ADAMTS4 immunolabelling in the MBH of DIO-Veh and DIO-STZ mice and (**E**) associated quantification. (**F**) Correlation between ME ADAMTS4 immunolabelling and blood glucose across multiple models, analysed by linear regression. (**G**) Working model linking hypoglycaemia-induced regulation of ME oligodendrocyte lineage cells, ADAMTS4 expression, blood-hypothalamic barrier remodelling and brain glucose sensing. All data presented as mean ± S.E.M., scale bars represent 100 μm. Data analysed using an unpaired student’s t-test- (B, C-E), (B, C, E) or linear regression (F), ∗*p* < 0.05, ∗∗*p* < 0.01.

To determine whether acute and chronic changes in blood glucose levels change ME ADAMTS4 expression, we first examined the effect of insulin-induced hypoglycaemia, a challenge which increases permeability across the blood-hypothalamus barrier [6]. We found a significant decrease in ME ADAMTS4 expression in brain tissues from mice treated with a bolus of insulin (Figure 8A–B), alongside a reduction in WFA + immunolabelling in the ME (Supplemental Fig. 7A–B), suggesting that ADAMTS4 expression responds to acute changes in peripheral glucose levels. We then used a model of diabetes, achieved through the combination of diet-induced-obesity (DIO) and streptozotocin (STZ) administration in C57BL/6J mice to induce insulin resistance and pancreatic β cell dysfunction. Compared to vehicle treated animals (DIO-Veh), treatment with STZ (DIO-STZ) increased ad libitum fed (Supplemental Fig. 7C) and fasting (Figure 8C) blood glucose and produced glucose intolerance (Supplemental Fig. 7D–E). Chronic hyperglycaemia in this model was associated with increased ADAMTS4 expression in the ME (Figure 8E–F). In addition, we found a strong correlation between blood glucose levels and ME ADAMTS4 expression (Figure 8G). Thus, changes in blood glucose levels regulate the expression of ADAMTS4 in the ME.

## 4 Discussion

Here we identify a role for ME oligodendrocytes in the central response to hypoglycaemia and in the activation of the counterregulatory response that restores glucose homeostasis. We show that changes in blood glucose levels regulate the density of newly formed oligodendrocytes in the ME and the expression of ADAMTS4, which regulates blood–hypothalamus barrier permeability, neuronal glucose sensing in the ARH, and the neuroendocrine control of blood glucose. We further find that ADAMTS4 expression is dysregulated in diabetic mice, identifying impaired blood–hypothalamus barrier function as a candidate mechanism contributing to fasting hyperglycaemia in diabetes.

A key finding of this study is that glycaemia acts as a physiological regulator of adult oligodendrogenesis in the ME. Hypoglycaemia increased the density of newly formed oligodendrocytes without altering OPC proliferation or total OPC number, consistent with enhanced survival or accelerated differentiation of premyelinating cells. In contrast, 2DG-induced hyperglycaemia increased OPC proliferation but reduced the density of newly formed oligodendrocytes, implying impaired differentiation or survival. This differs from our previous observation that refeeding promotes ME OPC differentiation [20]; postprandial hyperglycaemia in that context is accompanied by numerous other nutrient and hormonal changes that may collectively shape the OPC response. Moreover, 2DG-induced hyperglycaemia is more sustained and severe than the physiological postprandial rise, and the observed loss of newly formed oligodendrocytes may therefore reflect glucotoxicity. Collectively, these data position ME oligodendrogenesis as a rapid, bidirectionally regulated component of the central response to fluctuations in circulating glucose, extending previous work demonstrating structural plasticity of the ME in response to metabolic state [5].

How glucose controls ME oligodendrogenesis remains to be fully resolved, but our data argue against a cell-autonomous, metabolism-based mechanism. The opposite effects of hypoglycaemia and 2DG support this interpretation. Both challenges suppress intracellular glucose metabolism, yet they drive opposite changes in ME oligodendrogenesis. Because they produce opposite effects on extracellular glucose levels, this supports the conclusion that OPC fate decisions are governed by extracellular glucose availability, not by the rate of intracellular glucose catabolism. Recent data showing that the acetylation of aldolase C silences the aldolase–AMPK low-glucose-sensing pathway in OPCs, support this possibility [46]. This mechanism prevents low intracellular glucose levels from activating AMPK and permits OPC proliferation and differentiation to proceed even in low-glucose conditions, effectively disconnecting sensing of intracellular glucose deficit from cell fate decisions. Of note, oligodendrocyte lineage cells do not appear to possess the canonical machinery for glucose sensing: GLUT2, glucokinase and the KATP-channel subunit Kir6.2 are essentially absent from OPCs according to available single-cell transcriptomics datasets of the arcuate nucleus and median eminence [20,40], circumstantially supporting the interpretation that OPC sense glucose independently of intracellular glucose metabolism. Extracellular glucose levels might be relayed to OPCs through glucose-sensing tanycytes or neurons of the ME niche, or through circulating metabolites such as ketone bodies and lactate — to control the differentiation of ME OPCs into newly formed oligodendrocytes.

Interestingly, local glucose availability has been identified as a determinant of OPC behaviour, brain regions with higher glucose showing greater OPC proliferation [47]. In addition, regional differences in vascularisation generate local glucose gradients that drive region-specific OPC dynamics [47]. Thus, the blood–brain barrier properties of the ME, allowing local cells to experience the full dynamic range of systemic glycaemic fluctuations, provide an account of why ME oligodendrogenesis is acutely tuned to changes in blood glucose.

An important conceptual advance of this work is the dissociation between adult oligodendrogenesis and new myelin formation in metabolic regulation. Genetic blockade of new oligodendrocyte generation impaired glucose tolerance and the counterregulatory response to hypoglycaemia, whereas conditional Mbp deletion in adult OPCs — which prevents new compact myelin formation while preserving OPC differentiation — had no effect on glucose homeostasis. These findings argue that newly formed, premyelinating oligodendrocytes, rather than newly generated compact myelin, mediate the metabolic phenotype. Consistent with this, acute glycaemic manipulations did not alter ME myelin density. Although myelinated magnocellular axons traversing the ME have been implicated in glucagon regulation, neither glucagon secretion nor osmoregulatory responses were altered in Myrffl/fl mice, and whole-brain 3D imaging revealed that adult myelin plasticity was spatially restricted to the ME and did not extend along magnocellular projections. Together, these results support the emerging view that oligodendroglial lineage cells regulate neural circuit function through non-canonical mechanisms beyond saltatory conduction.

We further show that blockade of adult oligodendrogenesis remodels the blood–hypothalamus barrier, phenocopying key vascular changes observed during fasting, including increased vascular permeability and altered tanycytic paracellular organisation [5]. While previous work established tanycytes as mediators of ME vascular remodelling, our findings identify oligodendrocyte lineage cells as an additional regulatory component, suggesting that structural plasticity of the ME integrates coordinated inputs from glial, vascular and extracellular-matrix elements. Although our current data do not resolve how oligodendrocytes interact with other ME cell types including tanycytes and neurons, the identification of ADAMTS4 as a secreted effector (see below) provides a putative paracrine mechanism. The ME is uniquely positioned outside the classical blood–brain barrier, allowing circulating glucose to be sensed rapidly; by regulating barrier permeability, ME oligodendrogenesis may control the kinetics and amplitude of glucose access to arcuate circuits.

Our findings support a role for ME–ARH glucose sensing in the counterregulatory response to hypoglycaemia. In Myrffl/fl mice, the hyperglycaemic response to neuroglycopenia was accelerated and amplified, suggesting that the ME–ARH barrier normally gates the speed and amplitude of hypothalamic circuit activation. Traditionally, glucose sensing in the counterregulatory response has been attributed primarily to neurons in the ventromedial nucleus [34]. However, the ME–ARH region contains glucose-responsive AgRP neurons that influence the HPA axis via projections to PVH CRH neurons [48,49] and via direct projections to CRH terminals within the ME [50]. Given its anatomical exposure to the circulation, the ME may serve as an early detector of hypoglycaemia, recruiting rapid neuroendocrine responses before parenchymal sensors are engaged. One possibility is that ME sensors are activated first, with subsequent structural remodelling of the barrier permitting altered glucose access to deeper hypothalamic nuclei such as the VMN. The temporal dynamics of this remodelling remain to be defined and represent an important direction for future study.

Mechanistically, we identify ADAMTS4 as a candidate effector linking oligodendrogenesis to blood–hypothalamus barrier permeability, hypothalamic glucose sensing, and systemic glucose homeostasis. ADAMTS4 is selectively expressed within the oligodendrocyte lineage and enriched in newly formed oligodendrocytes; its expression is regulated by acute changes in glycaemia and is robustly reduced in Myrffl/fl mice. Adamts4 deletion phenocopied the vascular and barrier alterations observed in Myrffl/fl mice, consistent with a direct role for ADAMTS4 in blood–hypothalamus barrier regulation. In vitro, ADAMTS4 modulates angiogenesis via VEGF sequestration [51]; accordingly, VEGFA expression was elevated in the ME of Adamts4 knockout mice, and VEGF is known to be induced by hypoglycaemia in ME tanycytes [5], positioning ADAMTS4 as a candidate regulator of the ME neurovascular niche. We additionally show that ongoing oligodendrogenesis is required to maintain hypothalamic perineuronal nets. Because newly formed oligodendrocytes are themselves major contributors to the synthesis of PNN components [20], the reduction in ARH PNNs in Myrffl/fl mice most likely reflects depletion of these PNN-synthesising cells rather than loss of the PNN-degrading enzyme ADAMTS4; the concurrent reduction in ADAMTS4 is therefore best interpreted as a marker of the depleted oligodendrocyte-lineage population rather than its cause. Notably, intra-MBH injection of recombinant ADAMTS4 recapitulated the glycaemic and endocrine phenotype of Adamts4 deletion (elevated blood glucose, hypercorticosteronaemia, reduced insulin sensitivity) without producing overt changes in vascular fenestration. This dissociation indicates that ADAMTS4-dependent regulation of glucose sensing is not exclusively mediated through changes in vascular permeability, and implicates additional mechanisms engaged by pharmacological ADAMTS4. Given its potent aggrecanase activity [52] and the presence of aggrecan-containing PNNs in the ARH [53], excessive extracellular-matrix remodelling — and consequent disinhibition of PNN-ensheathed glucose-sensing neurons — is a likely contributor. Taken together, these observations suggest that physiological ADAMTS4 levels must be maintained within a narrow range to preserve both barrier integrity and circuit stability, with both loss and gain of function leading to hypothalamic dysregulation.

The stage-specific expression of *Adamts4* within the oligodendrocyte lineage further predicts that acute and chronic glycaemic perturbations regulate ME ADAMTS4 in opposite directions. Because *Adamts4* expression peaks in actively myelinating oligodendrocytes and is low in premyelinating BCAS1+ cells, acute hypoglycaemia — which increases BCAS1+ premyelinating oligodendrocytes without changing MBP — is expected to reduce total ME ADAMTS4. Conversely, chronic hyperglycaemia, which reduces production of premyelinating cells while the mature ADAMTS4-expressing pool persists, is expected to increase ME ADAMTS4 relative to premyelinating markers. This reconciles the apparently paradoxical relationships between blood glucose and BCAS1 (negatively correlated) and between blood glucose and ADAMTS4 (positively correlated).

Consistently, we find that ADAMTS4 expression is downregulated during acute hypoglycaemia and dysregulated in a mouse model of diabetes. Genome-wide association studies link ADAMTS4 variants to fasting insulin levels [54], and insulin has been shown to suppress ADAMTS4 expression in peripheral cells [55]. Our data extend these associations to the hypothalamus and raise the possibility that impaired oligodendrocyte-derived ADAMTS4 signalling contributes to fasting hyperglycaemia through altered barrier permeability and glucose sensing. Given the increasing recognition that blood–brain barrier dysfunction contributes to metabolic disease [56], it will be important to determine whether altered ME oligodendrogenesis and ADAMTS4 signalling play a causal role in obesity and diabetes, and whether restoration of this pathway can improve metabolic control.

Several limitations of this study warrant consideration. First, our conditional models rely on tamoxifen inducible Cre recombination, and tamoxifen can independently affect metabolism, brain physiology and glial biology [57]. However, both experimental and control animals received identical tamoxifen treatment, and the metabolic phenotype of Myrffl/fl mice was absent in tamoxifen-treated Mbpfl/fl mice — in which recombination occurs in the same Pdgfrα+ population — providing an internal control against tamoxifen-specific confounds. Second, our streptozotocin (STZ) model of chronic hyperglycaemia should be interpreted with caution, because STZ is taken up via GLUT2, which is expressed by tanycytes, and could therefore alter ME barrier properties independently of its diabetogenic action.

In conclusion, this work identifies adult oligodendrogenesis as a metabolically responsive and functionally essential component of hypothalamic barrier plasticity, expanding the role of oligodendrocyte lineage cells from passive support cells to active regulators of systemic metabolic homeostasis.

## CRediT authorship contribution statement

**Sophie Buller:** Writing – review & editing, Writing – original draft, Methodology, Investigation, Formal analysis, Data curation, Conceptualization. **Emily O. Staricoff:** Methodology, Investigation, Formal analysis, Data curation, Conceptualization. **Christine Riches:** Methodology, Investigation, Formal analysis, Data curation. **Anthony Tsang:** Investigation, Formal analysis, Data curation. **Giada Giavara:** Methodology, Investigation, Formal analysis, Data curation. **Masa Josipovic:** Investigation, Formal analysis. **Kentaro Ikemura:** Methodology, Investigation, Data curation. **Gabriel Opoku:** Investigation. **Ikumi Sato:** Investigation. **Satoshi Hirohata:** Investigation. **Saskia Stenzel:** Investigation. **Stuart G. Nayar:** Investigation. **Marta Ramos Vega:** Investigation, Formal analysis, Data curation, Conceptualization. **Jacob Hecksher-Sørensen:** Methodology, Investigation. **Sebastian Timmler:** Investigation, Formal analysis, Data curation. **Georgina K.C. Dowsett:** Formal analysis, Data curation. **Brian Y.H. Lam:** Investigation, Data curation. **Giles S.H. Yeo:** Conceptualization. **Kimberly M. Alonge:** Conceptualization. **Huiliang Li:** Investigation. **William D. Richardson:** Investigation. **Mark L. Evans:** Investigation. **Clemence Blouet:** Writing – review & editing, Writing – original draft, Visualization, Validation, Supervision, Resources, Project administration, Methodology, Investigation, Funding acquisition, Formal analysis, Data curation, Conceptualization.

## Funding

This work was supported by a 10.13039/501100000265Medical Research Council grant (MR/S011552/1; CB), a 10.13039/100010269Wellcome Trust PhD Studentship (108926/Z/15/Z; SB), 10.13039/501100000361Diabetes UK (22/0006401; CB), 10.13039/501100000265Medical Research Council Metabolic Disease Unit and Mouse Biochemistry Laboratory Grants (MC_UU_00014/5) and (MRC_MC_UU_12012/5), a 10.13039/100010269Wellcome Trust Strategic Award (208363/Z/17/Z), a 10.13039/100010269Wellcome Trust Investigator Award (108726/Z/15/Z; WDR), a 10.13039/100010269Wellcome Trust Investigator Award (214286/Z/18/Z; HL, WDR) and a Sanming Award (SZSM201911003; WDR, HL) from the Municipal Government of Shenzhen, China, a Grant-in-Aid for Scientific Research (A) from the 10.13039/501100001691Japan Society for the Promotion of Science (JSPS; 20H00548; SH) and a Grant-in-Aid for Early-Career Scientists from the JSPS (23K16796; IS). EOS, CR, JS and MLE received funding from the 10.13039/501100010767Innovative Medicines Initiative 2 Joint Undertaking (JU) under grant agreement No. 777460 (HypoRESOLVE). The JU receives support from the European Union’s Horizon 2020 research and innovation programme and EFPIA and T1D Exchange, JDRF, International Diabetes Federation (IDF), The Leona M. and Harry B. Helmsley Charitable Trust. The University of Cambridge has received salary support for MLE through the National Health Service in the East of England through the Clinical Academic Reserve. The manuscript reflects the authors’ views, and the funders are not responsible for any use that may be made of the information it contains. For the purposes of open access, the authors have applied a CC-BY public copyright license to any Author Accepted Manuscript version arising from this submission.

## Declaration of competing interest

The authors declare the following financial interests/personal relationships which may be considered as potential competing interests: Clemence Blouet reports financial support was provided by Diabetes UK. Clemence Blouet reports financial support was provided by Medical Research Council. Sophie Buller reports financial support was provided by Wellcome Trust. Bill Richardson reports financial support was provided by Wellcome Trust. HL reports financial support was provided by Wellcome Trust. Mark Evans reports financial support was provided by ERC. If there are other authors, they declare that they have no known competing financial interests or personal relationships that could have appeared to influence the work reported in this paper.

## Data Availability

Data will be made available on request.
