# Supplementary material for: Adaptive oligodendrogenesis regulates blood-hypothalamus barrier permeability, hypothalamic glucose sensing and systemic glucose homeostasis in male mice
Source: Mol Metab. 2026 Aug 6;112:102428. doi: 10.1016/j.molmet.2026.102428 (PMC13521147; doi:10.1016/j.molmet.2026.102428)
Supplement: Supplementary file 1 [file mmc1.docx]

**Supplemental Figure 1: Acute changes in glycaemia regulate oligodendrocyte lineage progression in the adult ME.** (**A**) Blood glucose levels in C57BL/6J mice one hour after the intraperitoneal administration of vehicle (saline; 10 ml/kg;n=7), 2-deoxy-d-glucose (2DG; 250 mg/kg;n=8), glucose (2 g/kg;n=8) or insulin (0.75 U/kg;n=10). (**B**) Quantification of oligodendrocyte lineage cells (SOX10^+^) in the ME one hour after vehicle, 2DG, glucose or insulin administration. (**C**) Representative images of Myelin basic protein (MBP), a myelin structural protein, in the MBH of mice that received vehicle or insulin and (**D**) associated quantification. All data presented as mean ± SEM, scale bars is 100 μm. Data analysed using one-way ANOVA with Dunnett’s multiple comparisons test (A,B) or an unpaired student’s t test when comparing 2 independent groups (D), ****p<0.0001.

**Supplemental Figure 2: Adult oligodendrocyte differentiation is required for glucose homeostasis**

(**A**) Blood glucose in *Myrf^+/+^* and *Myrf^fl/fl^* mice over 120 minutes after an intraperitoneal injection of vehicle (saline; 10 ml/kg) or 2-deoxy-d-glucose (2DG; 250 mg/kg) and (**B**) the resulting area under the curve. (**C**) Food intake over 180 minutes after vehicle or 2DG administration in *Myrf^+/+^* and *Myrf^fl/fl^* mice. (**D**) Plasma glucagon levels 30 minutes after vehicle or 2DG administration in *Myrf^+/+^* and *Myrf^fl/fl^* mice. Data presented as mean ± SEM. Data analysed by two-way ANOVA with Tukey’s (B, D) or Sidak’s multiple comparisons test (A, C).

**Supplemental Figure 3: Adult myelin generation is not required for the maintenance of glucose homeostasis.** (**A)** Representative images of arginine vasopressin immunolabelling in the mediobasal hypothalamus of myelin regulatory factor (MYRF) conditional knockout mice (*Myrf^fl/fl^*, n=6) and controls (*Myrf^+/+^*, n=6) and (**B)** associated quantification. (**C)** Daily water intake in *Myrf^fl/fl^* and *Myrf^+/+^* mice. (**D)** Plasma osmolarity in *Myrf^fl/fl^* and *Myrf^+/+^* mice at baseline and after 12 hours water deprivation. **(E**) Representative video of 3D imaged brain and **(F**) coronal maps of mGFP fluorescence in *Pdgfrα-CreERT2*;*taumGFP* mice dosed with tamoxifen at P60 and perfused at P90. Scale bar is 1 mm. Immunolabelling against (**G**) pan-oligodendrocyte marker SOX10, OPC marker PDGFRa and yellow fluorescent protein (YFP) and **(J)** Breast carcinoma amplified sequence 1 (BCAS1) in the ME of MBP conditional knockout mice (*Mbpfl/fl*, n=5) and controls (*Mbp^+/+^*, n=4) and associated quantifications of (**H**) oligodendrocyte lineage cells, (**I**) oligodendrocyte lineage cells expressing YFP (newly formed oligodendrocytes = newly formed Ols, SOX10+/PDGFRα-/YFP+) and (**K**) BCAS1 area fraction. (**L**) Blood glucose in *Mbp^+/+^* and *Mbpfl/fl* mice over 120 minutes after an intraperitoneal injection of vehicle (saline; 10 ml/kg) or 2-deoxy-d-glucose (2DG; 250 mg/kg) and (**M**) the resulting area under the curve. (**N**) Food intake over 180 minutes after vehicle or 2DG administration in *Mbp^+/+^* and *Mbpfl/fl* mice (n=8). Data presented as mean ± SEM, scale bars represent 100 μm. Data analysed by unpaired student’s t test (B, C, H, J, K) or two-way ANOVA with Tukey’s or Sidak’s multiple comparisons test (F, G, H, L), **p<0.01.

**Supplemental Figure 4: Blockade of adult oligodendrogenesis remodels the blood-hypothalamus barrier**. Representative images of (**A**) Vimentin and **(B)** GFAP immunolabelling in the mediobasal hypothalamus of myelin regulatory factor (MYRF) conditional knockout mice (*Myrf^fl/fl^*, n=6) and controls (*Myrf^+/+^*, n=8) and (**B, D**) associated quantification. Scale bars represent 100 μm. Data presented as mean ± SEM and analysed by unpaired students t-test.

Supplemental Figure 5: Adult oligodendrogenesis is required for the maintenance of hypothalamic perineuronal nets and ADAMTS4 expression. (A, B) Oligodendrocyte lineage population from HypoMap [25]. Colours indicated C66 clustering, and highlight 3 distinct oligodendrocyte populations, representative of OPC, NFOL and mature OL. (C) Heatmap displaying average log-normalized expression of marker genes of different stages of oligodendrocyte maturation in the 3 C66 oligodendrocyte clusters.

**Supplemental Figure 6: ADAMTS4 regulates the blood-hypothalamus barrier, hypothalamic glucose sensing and glucose homeostasis**. Representative images of **(A)** MBP and (**C**) BCAS1 immunolabelling in the ME of *Adamts4^-/-^* and *Adamts4^WT^* mice and (**B, D**) associated quantifications. Representative images of (**E**) laminin immunolabelling in the MBH of animals injected with aCSF or ADAMTS4 1 hour prior to sacrifice and (**F**) associated quantifications. Data presented as mean ± SEM and analysed by unpaired student’s t-test (D, F, H) or Mann-Whitney test (B), *p<0.05, ***p<0.001, ****p<0.0001, n=8-18/group. Scale bars represent 100 μm.

**Figure S7: Median eminence ADAMTS4 expression is regulated by glycaemia**

Representative images of (**A**) Wisteria floribunda agglutinin (WFA) lectin in the mediobasal hypothalamus (MBH) one hour after intraperitoneal (ip) administration of vehicle (saline; 10 ml/kg, n=7) or insulin (0.75 U/kg, n=7) to C57BL/6J mice and associated quantification (**B**). (**C**) *Ad libitum* fed blood glucose levels in diet-induced obese (DIO) C57BL/6J mice over 4 weeks following ip administration of vehicle (saline, 10 ml/kg; DIO-Veh) or streptozotocin (STZ; 100 mg/kg; DIO-STZ). (**D**) Blood glucose levels in DIO-Veh and DIO-STZ mice over 2 hours following ip administration of glucose (1 g/kg) and (**E**) associated area under the curve. All data presented as mean ± SEM, scale bars represent 100 μm. Data analysed by unpaired student’s t-test. *p<0.05, ***p<0.001.
